# Nanosensitizer for Cancer Radioimmunotherapy via Anti‐IL‐35 Blockade Boosted Innate Immunity Activation

**DOI:** 10.1002/advs.202504252

**Published:** 2025-06-26

**Authors:** Yinfei Zheng, Shuting Zheng, Yushu Liao, Zede Wu, Chenxi He, Qiuyu Li, Honglei Hu, Zheyu Shen, Yikai Xu, Chenggong Yan, Bingxia Zhao, Meirong Hou

**Affiliations:** ^1^ Department of Medical Imaging Center Nanfang Hospital Southern Medical University Guangzhou 510515 China; ^2^ Cancer Research Institute School of Basic Medical Sciences Southern Medical University Guangzhou 510515 China; ^3^ School of Biomedical Engineering Southern Medical University Guangzhou 510515 China; ^4^ Department of Radiology The Second Affiliated Hospital of Guangzhou Medical University Guangzhou 510260 China; ^5^ Experimental Education/Administration Center School of Basic Medical Science Southern Medical University Guangzhou 510515 China

**Keywords:** IL‐35 blockade, MR imaging, radioimmunotherapy, theranostics, tumor microenvironment

## Abstract

The cGAS‐STING signaling pathway has emerged as a promising target for cancer immunotherapy. However, STING agonists have a dual‐edged nature. Although they enhance antitumor T cell activity, STING agonists also elicit protumorigenic effects by promoting IL‐35‐producing B regulatory (Breg) cells, suppressing natural killer (NK) cell density, and fostering immune suppression. To address these challenges, this work develops a tumor microenvironment‐responsive hollow mesoporous nanosystem that degrades under high glutathione conditions, thereby releasing the STING agonist MSA‐2 and Mn ions. This nanosystem facilitates magnetic resonance imaging‐guided chemodynamic therapy and radiosensitization, efficiently activating the cGAS‐STING pathway by disrupting mitochondrial and nuclear DNA. Notably, by integrating this nanosystem with an anti‐IL‐35 blockade, this work successfully mitigates the Breg cell‐mediated suppression of NK cells, restores innate immune responses, and enhances antitumor efficacy. This study highlights the pivotal role of anti‐IL‐35 in reversing immunosuppression, enhancing innate immunity, and establishing a synergistic theranostic platform for molecular imaging‐guided cancer radioimmunotherapy.

## Graphical abstract

Zheng et al. designed a tumor microenvironment (TME)‐responsive nanosystem, HmBMH@MSA‐2. It targets tumors, releases MSA‐2 and Mn ions in high glutathione (GSH) conditions, achieving STING agonist delivery and MRI. The nanosystem promotes chemodynamic therapy (CDT), achieves radiosensitization, and activates the cGAS‐STING pathway. Combined with anti‐IL‐35 therapy, it neutralizes IL‐35 from B regulatory cells, restores natural killer (NK) cell antitumor activity. This strategy reverses the tumor‐immunosuppressive microenvironment and boosts innate immunity activation.

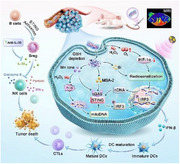

## 1 Introduction

The immune system plays a pivotal role in tumor prevention and suppression and significantly influences the progression of traditional cancer therapies.^[^ ^1^ ^]^ Cancer immunotherapy is based on the recognition and interaction between immune cells and specific signaling molecules associated with tumorigenesis. Notably, the cyclic GMP‐AMP synthase (cGAS)‐stimulator of interferon genes (STING) signaling pathway has emerged as a promising target for cancer immunotherapy.^[^ ^2^, ^3^ ^]^ The accumulation of tumor‐derived double‐stranded DNA (dsDNA) in the cytoplasm triggers the cGAS sensor to produce cyclic GMP‐AMP (cGAMP), which in turn activates the STING protein.^[^ ^4^, ^5^ ^]^ Following this, STING stimulates the transcription of genes involved in the innate immune response, resulting in the synthesis of type I interferons (IFN‐I), which activate a robust antitumor immune response.^[^ ^6^, ^7^ ^]^

Numerous methods have been investigated and validated for the activation of the cGAS‐STING signaling pathway. Specifically, radiotherapy (RT) induces cancer cell death and dsDNA breaks through ionizing radiation, thereby activating the cGAS‐STING signaling pathway and enhancing antitumor immunity.^[^ ^8^ ^]^ Nevertheless, challenges such as radiation tolerance significantly decrease the efficacy of RT alone for tumor cell eradication.^[^ ^9^ ^]^ Additionally, the use of STING agonists represents a straightforward approach for activating the cGAS‐STING signaling pathway. In numerous preclinical models, pharmacological activation of STING has been an effective immunotherapeutic strategy for cancer.^[^ ^10^ ^]^ However, STING agonists are double‐edged swords. The widespread presence of STING in the body means that when most STING agonists are administered systemically, they can lead to undesirable side effects and have a brief duration of action within the bloodstream.^[^ ^11^ ^]^ This significantly reduces their therapeutic potential. However, the recent surge in nanotechnology has opened up promising new paths to address these challenges. Targeted nanocarriers can solve these problems by improving drug delivery precision, lowering systemic toxicity and extending the half‐life.^[^ ^12^, ^13^ ^]^ Previously, we developed an innovative manganese‐bismuth nanosystem‐based synergistic strategy for glutathione (GSH)‐responsive drug release and the catalysis of O_2_ production from H_2_O_2_, thereby alleviating tumor hypoxia. Additionally, this strategy leverages the high‐Z element deposition of X‐rays to synergistically augment RT efficacy.^[^ ^14^ ^]^

However, activation of cGAS‐STING in the tumor microenvironment (TME) may result in further complications. Li et al. found that, in contrast to the antitumor effect of STING agonists on T cells, in B cells, STING agonists promote tumorigenesis by increasing the population of IL‐35‐producing B regulatory (Breg) cells within tumors.^[^ ^15^ ^]^ B cells constitute a significant fraction of the TME,^[^ ^16^ ^]^ and STING activation within B cells induces their differentiation into Breg cells.^[^ ^17^, ^18^ ^]^ Breg cells secrete cytokines, including IL‐10, IL‐35, and transforming growth factor‐beta (TGF‐β).^[^ ^19^, ^20^ ^]^ Notably, IL‐35 plays a crucial role in the interactions between malignant cells and surrounding immune cells in the TME, creating an immunosuppressive environment and impeding effective anti‐tumor immune responses.^[^ ^21^, ^22^ ^]^ Importantly, IL‐35 from Breg cells suppresses natural killer (NK) cell function by inhibiting proliferation, reducing granzyme B and TRAIL expression, and modulating signaling pathways.^[^ ^15^, ^17^ ^]^ NK cells, essential components of the innate immune system and the first line of immune defense, can detect and eliminate cancer cells early through their unique recognition mechanisms.^[^ ^23^ ^]^ A decreased NK cell density weakens the ability to control tumor growth and metastasis and increases the risk of recurrence.^[^ ^24^ ^]^ Blocking IL‐35 reverses these effects by upregulating the PI3K/AKT/mTOR and glycolysis pathways, enhancing NK cell proliferation and cytotoxicity against tumor cells. This combination with STING agonists strategy has shown promise to effectively inhibit tumor growth, presenting a novel therapeutic approach for clinical oncology.^[^ ^15^ ^]^

**Scheme 1:**
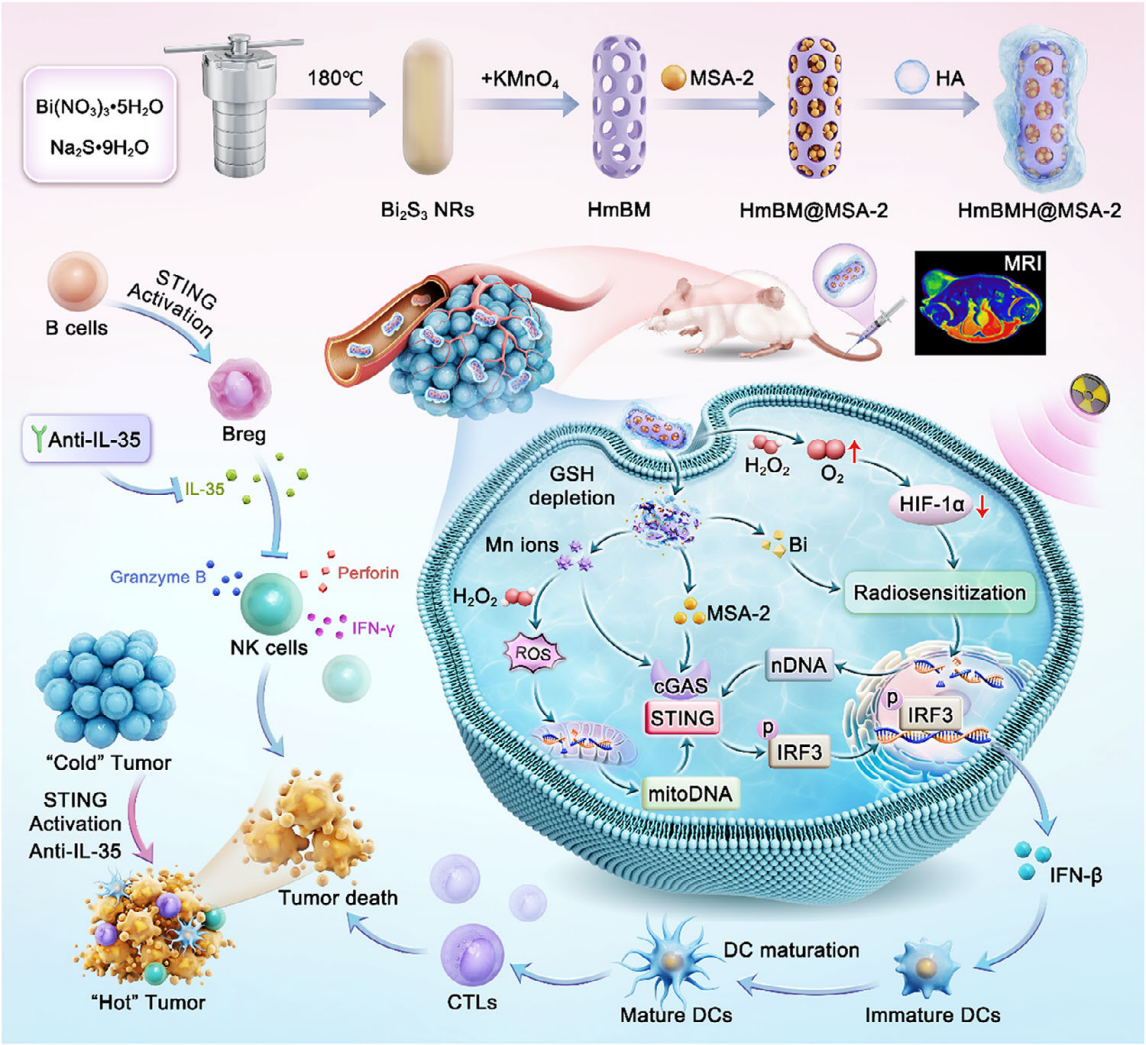
The mechanism of radioimmunotherapy enhancement based on HmBMH@MSA‐2 with anti‐IL‐35 blockade therapy.

Based on these considerations, we constructed TME‐responsive hollow mesoporous shell‐like nanorods (HmBM) to enhance radioimmunotherapy (**Scheme** **1**). These nanorods encapsulated a novel STING agonist, MSA‐2, and were further coated with hyaluronic acid (HA), resulting in hollow mesoporous BiMn‐HA@MSA‐2 (abbreviated as HmBMH@MSA‐2). The HmBMH@MSA‐2 nanosystem targeted tumor sites through CD44 receptors, underwent degradation under high GSH conditions in the TME, released MSA‐2 and Mn ions, and achieved targeted delivery of STING agonists and T1‐weighted magnetic resonance imaging (MRI). Additionally, the nanosystem facilitated the breakdown of intratumoral hydrogen peroxide into highly reactive hydroxyl radicals (·OH) through a Fenton‐like reaction, thereby inducing oxidative stress on mitochondria and prompting the release of mitochondrial DNA (mitoDNA) into the cytoplasm. Furthermore, it sensitized cancer cells to RT by alleviating hypoxia and depositing X‐rays with the high‐atomic‐number element Bi, thereby augmenting nuclear DNA (nDNA) damage. Consequently, Mn ions, MSA‐2, damaged mitoDNA and nDNA together enhanced the activation of cGAS‐STING signaling pathway, thereby leading to enhanced dendritic cell (DC) and T cell activation. Finally, the combination with anti‐IL‐35 blockade therapy neutralized the IL‐35 secreted by Breg cells that differentiated in response to STING activation, effectively restoring the antitumor activity of NK cells. This strategy offers a promising solution for reversing the tumor‐immunosuppressive microenvironment and boosting the activation of innate immunity.

## 2 Results and Discussion

### 2.1 Synthesis and Characterization of HmBMH@MSA‐2

**Figure 1:**
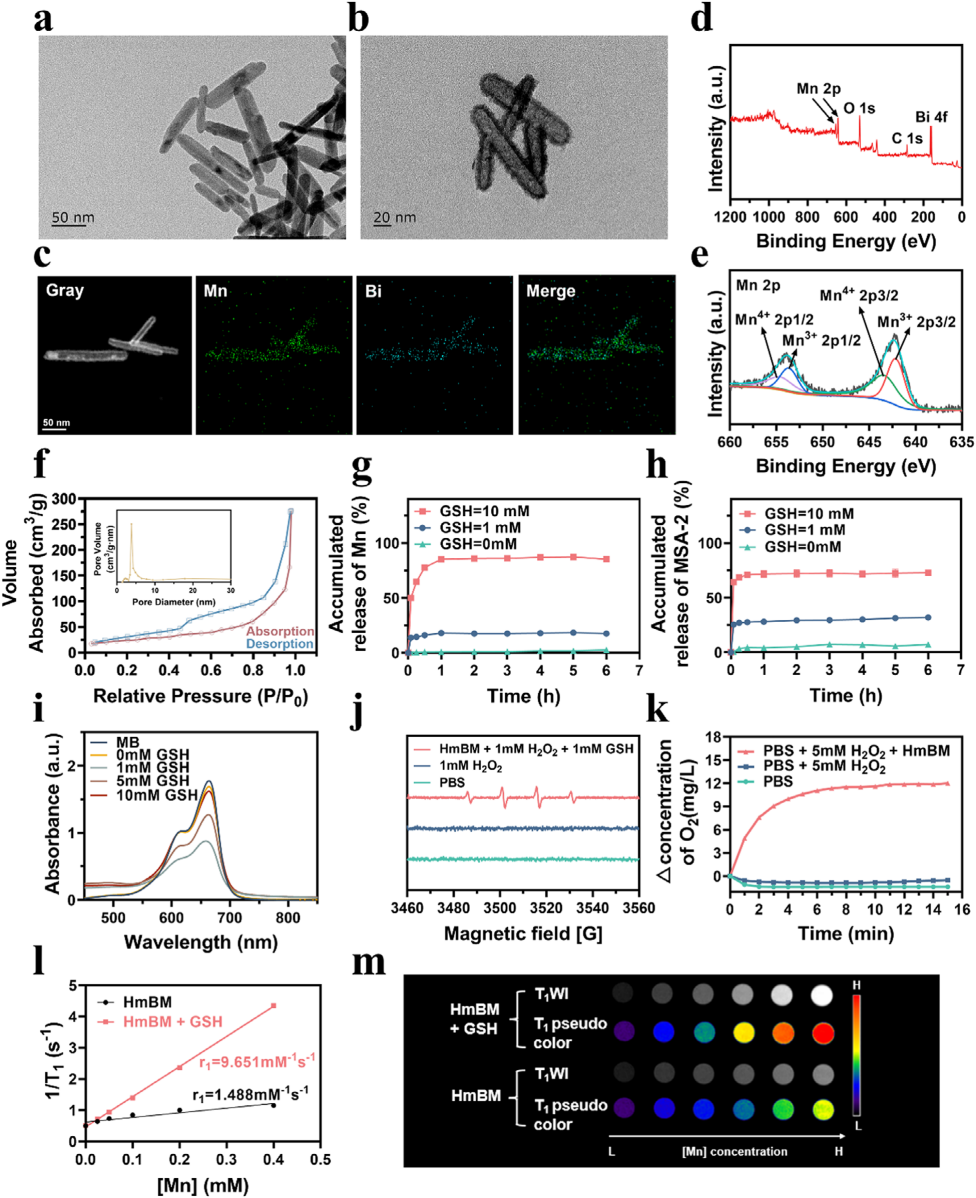
Characterization of HmBMH@MSA‐2. Representative TEM images of a) Bi_2_S_3_, b) HmBM. c) EDS elemental mapping of HmBM. d) XPS image of HmBM. e) High‐resolution XPS spectra of Mn 2p of HmBM. f) Nitrogen adsorption‐desorption isotherms and pore size distribution of HmBM. g) Mn components released from HmBM in different concentrations of GSH solution. h) The cumulative MSA‐2 release profile of HmBM@MSA‐2 in different concentrations of GSH solution. i) The UV–vis absorption spectra of MB after degradation by GSH treated HmBM‐mediated Fenton‐like reaction. j) EPR spectra of HmBM measured using a 2,2,6,6‐tetramethylpiperidine probe. k) O_2_ concentration variations of PBS solutions (pH = 7.4) mixed with different solvent. l) The longitudinal relaxation r1 values of HmBM in different solutions and m) the corresponding T1‐weighted MRI. Data were given as mean ± S.D. (n = 3).

Bi_2_S_3_ nanorods (NRs) were synthesized based on a previously reported method and served as sacrificial templates for the subsequent experiments (**Figure** **1**a).^[^ ^25^ ^]^ As shown in Figure 1b, nanorods with hollow mesoporous shell‐like structures were formed at room temperature by simply adding KMnO_4_ to the Bi_2_S_3_ NRs solution under stirring. Transmission electron microscopy (TEM) revealed a unique hollow shell‐like structure for HmBM, with an average long diameter of 86.2 ± 19.0 nm and an average cross diameter of 21.3 ± 3.7 nm (Figure S1, Supporting Information). High‐angle annular dark‐field scanning TEM‐based EDS elemental mapping revealed a uniform distribution of Mn and Bi (Figure 1c). Given the crucial role of the Mn valence state in GSH‐depleted Fenton‐like reactions and activatable MRI,^[^ ^26^, ^27^ ^]^ the X‐ray photoelectron spectroscopy (XPS) spectra of HmBM was analyzed. XPS revealed typical peaks associated with C 1s, O 1s, Bi 4f, and Mn 2p in HmBM (Figure 1d). As shown in Figure 1e, the high‐resolution Mn 2p XPS spectrum revealed that Mn primarily existed in the Mn^3+^ (Mn^3+^ 2p3/2: 642.11 eV and Mn^3+^ 2p1/2: 653.64 eV) and Mn^4+^ (Mn^4+^ 2p3/2: 643.37 eV and Mn^4+^ 2p1/2: 654.57 eV) states. Moreover, the Brunauer–Emmett–Teller (BET) surface area and average pore diameter of HmBM were 85.576 m^2^ g^−1^ and 3.819 nm, respectively (Figure 1f). This confirmed that the synthesized nanorods exhibited a mesoporous structure, which is suitable for antitumor drug loading.

MSA‐2 loading was determined by ultraviolet‐visible (UV–vis) scanning spectroscopy, ^[^ ^13^, ^28^ ^]^ with the characteristic peak of MSA‐2 at 322 nm (Figure S2, Supporting Information). The MSA‐2 drug loading capacity was determined to be 12.4% (Figure S3, Supporting Information). Hyaluronic acid (HA) was coated onto the surface of HmBM@ MSA‐2 to facilitate targeted delivery to CD44 receptors on the tumors. ^[^ ^29^ ^]^ HA modification decreased the surface zeta potential and increased the particle size (Figure S4, Supporting Information). Fourier transform infrared (FTIR) spectroscopy revealed a typical signal at 1038 cm^−1^, which was attributed to the saccharide group (Figure S5, Supporting Information),^[^ ^30^ ^]^ confirming successful HA modification.

Given that the ‐Mn‐O‐ bonds in Mn‐based nanostructures can be decomposed by elevated GSH levels in the TME,^[^ ^31^ ^]^ we further investigated the GSH‐triggered Mn ions and drug release profiles across various solutions. As expected, GSH consumption increased with increasing HmBM concentration (Figure S6, Supporting Information). Mn ions released from HmBM treated with and without GSH were monitored by ICP‐OES (Figure 1g). In the absence of GSH, only a minimal amount of Mn ions was released during the first 6 h. Conversely, in the presence of GSH, Mn ions release increased proportionally with GSH concentration, indicating that GSH enhanced the biodegradability of HmBM. Additionally, to investigate whether degradation of the nanosystem could further accelerate the release of the immobilized MSA‐2, HmBM@ MSA‐2 was subjected to identical conditions. As depicted in Figure 1h, the incorporation of GSH led to a significant MSA‐2 release from HmBM@MSA‐2. At 10 mM GSH, the cumulative MSA‐2 release reached ≈72.7% after 6 h. These findings suggest that HmBM has potential as a GSH‐ responsive drug delivery nanoplatform.

To investigate the chemodynamic therapy (CDT) effect, methylene blue (MB) was selected as an indicator for ⋅OH generation by HmBM. As shown in Figure 1i and Figure S7, Supporting Information, as the GSH concentration increased from 0 to 10 mM, MB in the NaHCO_3_/CO_2_ buffer containing H_2_O_2_ and GSH‐treated HmBM exhibited a notable color change. This suggests that Mn ions released from HmBM reacted with H_2_O_2_ in the presence of GSH, which could act as catalysts to drive the Fenton‐like reaction to produce ⋅OH. However, excess GSH (5 and 10 mM) inhibited the degradation of MB. The generation of ·OH was also determined by electron paramagnetic resonance (EPR), with a typical 1:2:2:1 signal observed in the HmBM + H_2_O_2_ and GSH group (Figure 1j). Furthermore, to test whether HmBM could decompose H_2_O_2_ into H_2_O and O_2_, a dissolved oxygen meter was used to monitor the generation of O_2_ (Figure 1k). With the addition of HmBM, oxygen production increased over time, demonstrating that HmBM accelerated H_2_O_2_ decomposition to generate O_2_ in the TME, which is crucial for alleviating tumor hypoxia. These results indicate that HmBM could serve as an effective agent for GSH depletion, thereby enhancing Mn ions‐mediated CDT efficacy.

To evaluate the GSH‐responsive MRI contrast enhancement effect, T1‐weighted images of HmBM were scanned in solutions with or without GSH. As presented in Figure 1l,m, the T1‐weighted MRI intensity considerably increased upon incubation with GSH and the relaxation coefficient (r_1_) increased from 1.488 to 9.651 mM^−1^s^−1^. These results demonstrate that Mn ions were released from HmBM in the presence of GSH, thus confirming the GSH‐mediated contrast enhancement potential of HmBM for T1‐weighted MRI.

### 2.2 CDT‐Mediated mitoDNA Damage and Release

**Figure 2:**
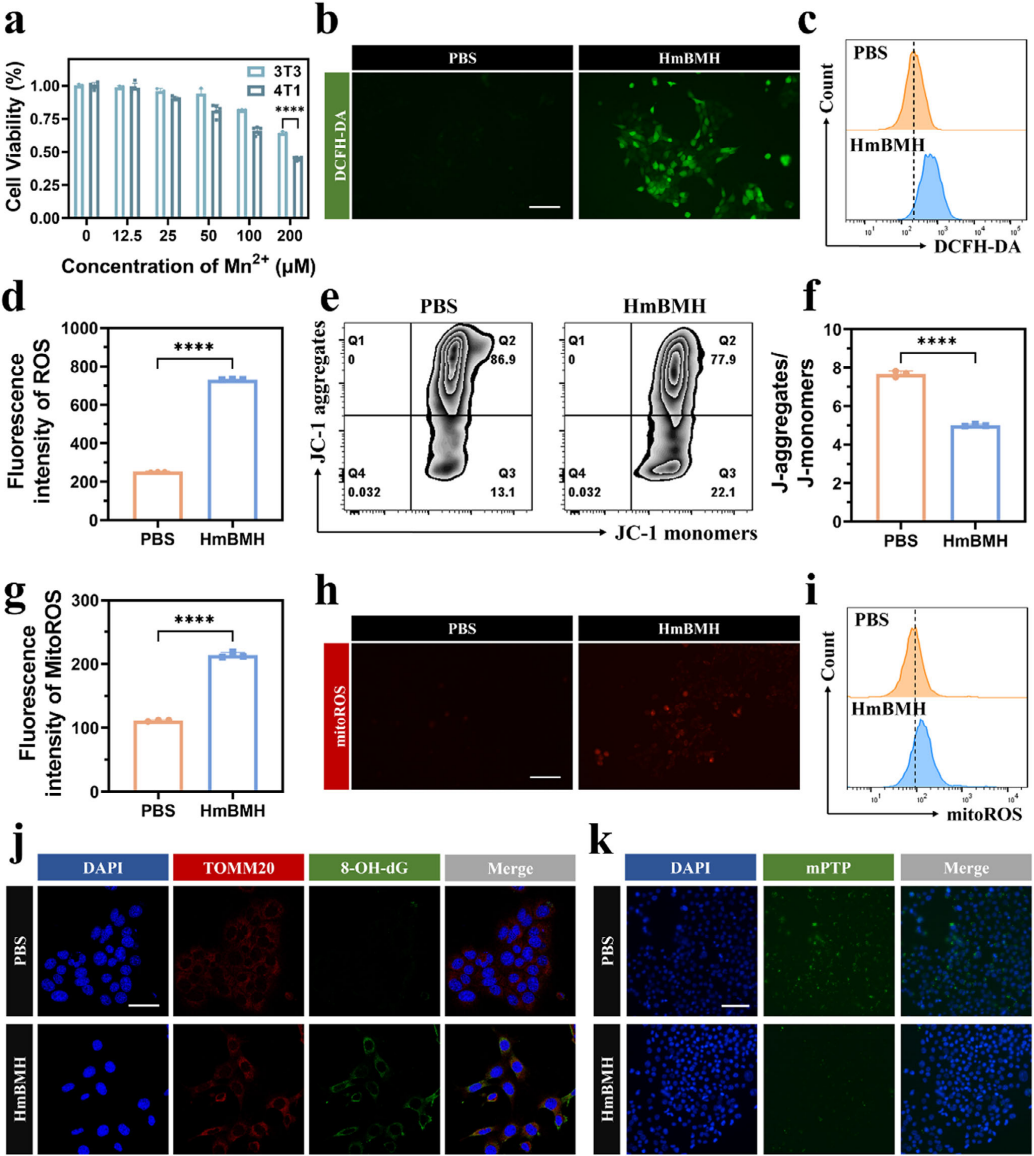
CDT‐mediated mitoDNA damage and release. a) The cell viability of 3T3 cells and 4T1 cells after co‐incubated with HmBMH for 24 h. b–d) ROS content in 4T1 cells observed and measured by inverted microscopy and flow cytometry after treatment with HmBMH, scale bar: 500 µm. e,f) Flow cytometry results of JC‐1 level in treated 4T1 cells. g–i) Intracellular mitochondrial superoxide intensity in 4T1 cells observed and measured by inverted microscopy and flow cytometry after treatment with HmBMH, scale bar: 500 µm. j) Representative immunofluorescence images showing mitoDNA oxidation levels in treated 4T1 cells stained with TOMM20 (red), 8‐OHdG (green), and DAPI (blue), scale bar: 50 µm. k) mPTP images of treated 4T1 cells observed by inverted fluorescence microscopy, scale bar: 500 µm. Data were given as mean ± S.D. (n ≥ 3). Statistical significance was calculated via Student t test and one‐way ANOVA with Tukey's test: ****p < 0.0001.

Encouraged by the satisfactory properties of HmBM, the in vitro cytotoxicity of HmBMH in MC3T3‐E1 normal cells and 4T1 tumor cells was evaluated using the CCK‐8 assay. As shown in **Figure** **2**a, the viability of MC3T3‐E1 cells was maintained above 80% when the concentration was less than 100 µM Mn. When reached 200 µM Mn, it had a slight effect on the viability of MC3T3‐E1 cells, but 4T1 cells exhibited reduced viability under the same Mn concentration. These phenomena may be attributed to the preferential targeting of tumor cells overexpressing the CD44 receptors by the HA‐conjugated nanosystems.^[^ ^32^, ^33^, ^34^ ^]^ The expression of the CD44 receptor on 4T1 and MC3T3‐E1 cells were confirmed using a western blot (Figure S8, Supporting Information). Subsequently, the cellular uptake of rhodamine B‐labeled HmBMH (RhB‐HmBMH) to investigate its active‐targeting ability via CD44‐receptor‐mediated endocytosis. Excess free HA was used to block CD44‐mediated cellular internalization. As shown in Figure S9, Supporting Information, after 3 h of incubation with RhB‐HmBMH, 4T1 cells pretreated with excess free HA exhibited weaker fluorescence than those without free HA. Similarly, only a few RhB‐HmBMH nanosystems were internalized by the MC3T3‐E1 cells owing to insufficient CD44 expression. These results indicate that HmBMH was specifically internalized by 4T1 cells through overexpressed CD44 receptors. We also verified the cellular internalization of HmBMH@MSA‐2, as encapsulation may affect the uptake efficiency. We compared the cellular uptake of HmBMH and HmBMH@MSA‐2 (Figure S10, Supporting Information). The results showed that the cellular uptake efficiency of HmBMH@MSA‐2 was comparable to that of HmBMH, indicating that the encapsulation process does not hinder cellular internalization.

Furthermore, the specific killing ability of HmBMH toward tumor cells may be attributed to the elevated GSH concentration in tumor cells, which triggers the CDT effect for selective tumor cell killing. To further test this hypothesis, a 2′,7′‐dichlorofluorescein diacetate (DCFH‐DA) probe, which becomes oxidized by intracellular ROS and subsequently emits green fluorescence, was used to monitor intracellular ROS production. As shown in Figure 2b, after incubation with HmBMH for 6 h, significant green fluorescence was observed in 4T1 cells stained with DCFH‐DA. Intracellular ROS levels were quantified by flow cytometry. The ROS level in the HmBMH group was 2.95‐fold higher than that in the PBS group (Figure 2c,d). Therefore, GSH‐rich environments could activate the CDT effect induced by HmBMH for tumor‐specific therapy.

Excessive ROS production induces mitochondrial dysfunction. Mitochondria are the primary sites for ROS production, and mitochondrial damage can further impair oxidative phosphorylation and the electron transport chain, leading to increased ROS production and release from the mitochondria.^[^ ^35^ ^]^ To investigate whether HmBMH could induce mitochondrial damage, a JC‐1 probe was used to measure the mitochondrial membrane potential (MMP). The JC‐1 probe exhibits a shift in fluorescence from red to green upon a decrease in MMP. JC‐1 typically aggregates on normal mitochondrial membranes with a relatively high MMP, but forms monomers on abnormal mitochondrial membranes with low MMP. Flow cytometry data in Figure 2e,f showed a decrease in the aggregate proportion and an increased monomer proportion in the HmBMH group compared to those in the PBS group, indicating MMP abnormality. Encouraged by the above results, we further investigated intramitochondrial ROS using a mitoSOX probe (red). As expected, cells treated with HmBMH exhibited strong red fluorescence (Figure 2g–i), suggesting that enhanced CDT may induce mitochondrial dysfunction and subsequently generate significant levels of mitochondrial ROS (mitoROS).

MitoDNA is susceptible to oxidative damage resulting from ROS generated in the mitochondrial matrix, coupled with the absence of protective histones.^[^ ^36^ ^]^ The effects of HmBMH on oxidized mitochondrial DNA (Ox‐mitoDNA) damage in 4T1 cells were assessed using confocal laser scanning microscopy (CLSM) after staining the Ox‐mitoDNA and mitochondria. In the HmBMH group, strong green fluorescence, indicative of Ox‐mitoDNA, was observed (Figure 2j, Figure S11, Supporting Information). The red fluorescence of TOMM20 co‐localized with the green fluorescence of 8‐OHdG, suggesting that the majority of the oxidative damage occurred in mitoDNA. Mitochondrial permeability transition pores (mPTP) facilitate mitoDNA release. Therefore, an mPTP assay kit was used to assess the impact of HmBMH on the opening state of mPTP. Upon triggering the opening of mPTP, calcein (green fluorescence) within the mitochondria is released into the cytoplasm and quenched by CoCl_2_, resulting in a reduction in intracellular green fluorescence. CLSM revealed a decrease in green fluorescence in the HmBMH group compared to that in the control group (Figure 2k, Figure S12, Supporting Information). This demonstrates that HmBMH significantly increased intracellular ROS levels, induced oxidative damage to mitochondria, and led to the release of Ox‐mitoDNA into the cytoplasm through the opening of mPTP. This process activated multiple immunostimulatory DNA sensors, including cGAS, thereby triggering an innate immune response.^[^ ^37^ ^]^

### 2.3 Radiosensitization‐Mediated nDNA Damage and Release

**Figure 3:**
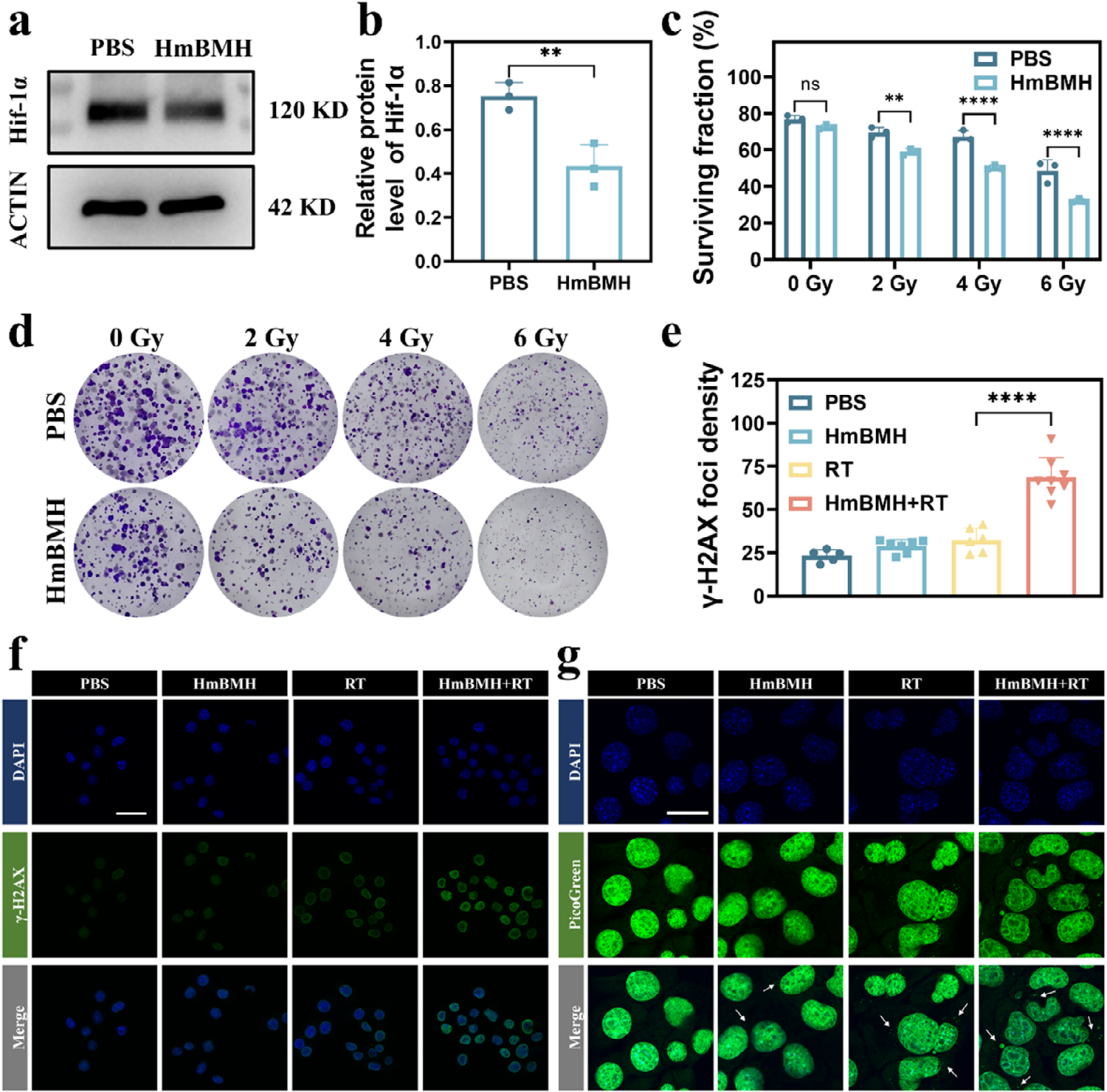
Radiosensitization‐mediated nDNA damage and release. a,b) Western blot analysis of HIF‐1𝛼 expression level in 4T1 cells in different groups. c) Clonogenic survival fraction of 4T1 cells after various treatments and exposed with X‐ray at different doses. d) Representative digital photos of clonogenic assay of 4T1 cells with different treatments from three biologically independent samples. e) Quantitative analysis of γ‐H2AX foci density. f) Representative immunofluorescence images of DNA damage marker γ‐H2AX in 4T1 cells with different groups followed by X‐ray irradiation (4 Gy). Green: γ ‐H2AX foci; Blue: cell nuclei. Scale bar: 50 µm. g) Representative images of picogreen‐stained 4T1 cells after treatment with HmBMH, scale bar: 25 µm. Data were given as mean ± S.D. (n ≥ 3). Statistical significance was calculated via Student t test and one‐way ANOVA with Tukey's test: **p <0.01, ****p < 0.0001.

RT is a common therapeutic strategy in clinical oncology that damages nDNA and releases it into the cytoplasm to activate the cGAS‐STING signaling pathway. However, only low levels of ROS can be generated at the recommended radiation dose to induce oxidative stress owing to insufficient X‐ray deposition within the tumor tissue and the prevalence of a hypoxic TME.^[^ ^38^, ^39^ ^]^ In order to achieve radiosensitization, relief of hypoxia and increased deposition of X‐rays at the tumor site are necessary. Encouraged by the satisfactory oxygen production performance of HmBMH, we then examined the effect of hypoxia alleviation at the cellular level by the hypoxia marker Hif‐1α. As shown in **Figure** **3**a,b, Hif‐1α expression was significantly lower in the HmBMH group than that in the PBS group. These findings suggest that HmBMH can effectively alleviate the hypoxic tumor microenvironment, which is expected to achieve radiosensitization. Subsequently, a clonogenic cell survival assay was performed to assess the ability of HmBMH to sensitize 4T1 breast cancer cells to RT. Briefly, 4T1 cells were irradiated with different X‐ray doses (0, 2, 4, and 6 Gy) with or without the addition of HmBMH. As expected, 4T1 cells incubated with HmBMH + X‐ray radiation showed a considerable reduction in the surviving fraction when compared with the control group (Figure 3c,d). This may be because HmBMH contains high‐Z element Bi, which can effectively deposit X‐rays as a radiosensitizer.

Cell radiosensitivity was achieved via the induction of DNA damage by RT. We measured the expression level of γ‐H2AX, a biomarker of dsDNA breaks, which is highly expressed in the DNA repair process and reflects DNA damage to some extent. Consistent with the above results, the HmBMH + X‐ray radiation group presented a higher level of DNA damage than the other groups (Figure 3e,f). These findings confirmed that HmBMH could act as an effective radiosensitizer to enhance DNA damage induced by RT, thereby effectively inhibiting the proliferation of tumor cells. Since RT promotes the release of dsDNA from the nucleus, and the release of damaged nDNA into the cytoplasm activates the cGAS‐STING signaling pathway,^[^ ^40^, ^41^ ^]^ we used Picogreen, a specific DNA‐binding fluorescent dye (green fluorescence), to detect cytosolic dsDNA that had escaped from the cell nucleus. As depicted in Figure 3g, extranuclear green fluorescence was observed in the HmBMH + X‐ray radiation group, indicating dsDNA accumulation in the cytoplasm. This observation indicated that HmBMH + X‐ray radiation treatment has the potential to trigger nDNA release. Overall, the above results suggest that HmBMH could sensitize RT by alleviating hypoxia, and the deposition of X‐rays caused nDNA cross‐linking damage. In addition, we assessed the extent of mitoDNA oxidation damage in 4T1 cells after treatment with RT alone, HmBMH alone, and the combination of RT and HmBMH by measuring Ox‐mitoDNA and mitochondrial staining. As shown in Figure S13, Supporting Information, the green fluorescence in the HmBMH+RT group was significantly stronger than that in the other groups, indicating that our nanosystems, when used in combination with RT, can also enhance the oxidative damage to mitoDNA.

### 2.4 HmBMH@MSA‐2 Enhanced Radioimmunotherapy

**Figure 4:**
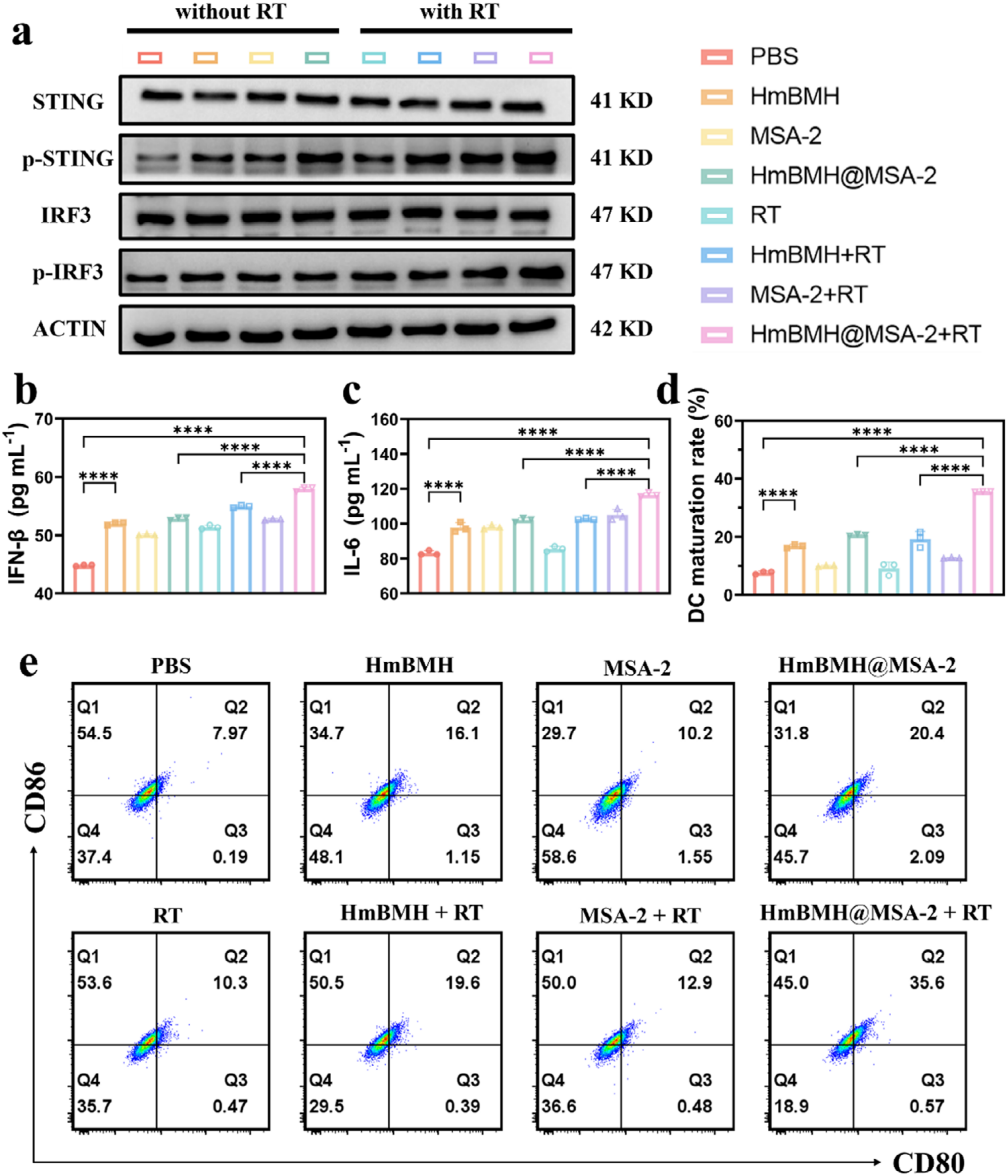
HmBMH@MSA‐2 enhanced radioimmunotherapy. a) Western blot analysis for proteins related to the cGAS/STING signaling pathway. ELISA results of b) IFN‐𝛽 and c) IL‐6 in the medium supernatant of 4T1 cells under different treatments. d) Statistical analysis and e) flow cytometry images of BMDCs maturation under different treatments. Data were given as mean ± S.D. (n = 3). Statistical significance was calculated via one‐way ANOVA with Tukey's test: ****p < 0.0001.

In cancer immunoregulation, the cGAS‐STING signaling pathway is initiated by the recognition of dsDNA in the cytoplasm to evoke innate immunity.^[^ ^42^ ^]^ In this study, HmBMH induced a Fenton‐like reaction, which caused mitochondrial oxidative stress‐induced mitoDNA damage. In addition, HmBMH sensitized RT to enhance nDNA damage. Furthermore, HmBMH served as a targeted drug‐carrying nanoplatform for the loading of the STING agonist MSA‐2, thereby enhancing immunotherapy. To ascertain whether HmBMH@MSA‐2 + RT activates innate immunity mediated by the cGAS‐STING signaling pathway, we analyzed the expression of STING pathway‐associated proteins using western blotting. HmBMH@MSA‐2 combined with RT significantly elevated the expression of key phosphorylated proteins (p‐STING and p‐IRF3) in the STING pathway (**Figures** **4**a, and S14, Supporting Information). We also established three groups for western blotting to assess the expression of p‐STING protein: RT alone, RT + HmBMH+MSA‐2 mixture, and RT + HmBMH@MSA‐2 nanosystem. As shown in Figure S15, Supporting Information, compared with the RT + HmBMH+MSA‐2 mixture group, the RT + HmBMH@MSA‐2 group exhibited higher levels of p‐STING protein expression. This indicates that the nanosystem effectively enhanced the cellular delivery efficiency of the components through the HmBMH targeting system. Additionally, the levels of IFN‐𝛽 and interleukin 6 (IL‐6) in the supernatant of 4T1 cells from various treatment groups were measured using ELISA kits. As expected, the HmBMH@MSA‐2+RT group significantly induced the release of IFN‐𝛽 and IL‐6 compared to the other groups (Figure 4b,c). These effects may be attributed to the leakage of mitoDNA and nDNA resulting from HmBMH@MSA‐2 + RT and the responsive release of the STING agonist MSA‐2 and Mn ions in the TME. Collectively, these factors activated the cGAS‐STING signaling pathway.

To investigate the capacity of HmBMH@MSA‐2 + RT to promote DC maturation in vitro, we used flow cytometry to detect the changes in the expression levels of CD80 and CD86. The flow cytometry results revealed that the HmBMH@MSA‐2+RT group exhibited the most pronounced effect on DC maturation compared to the other groups (Figure 4d,e). This effect may be attributed to the activation of the cGAS‐STING signaling pathway in the combined treatment group, resulting in significant up‐regulation of IFN‐I and subsequent induction of DC maturation.

### 2.5 In Vivo MR Imaging and Antitumor Effect

**Figure 5:**
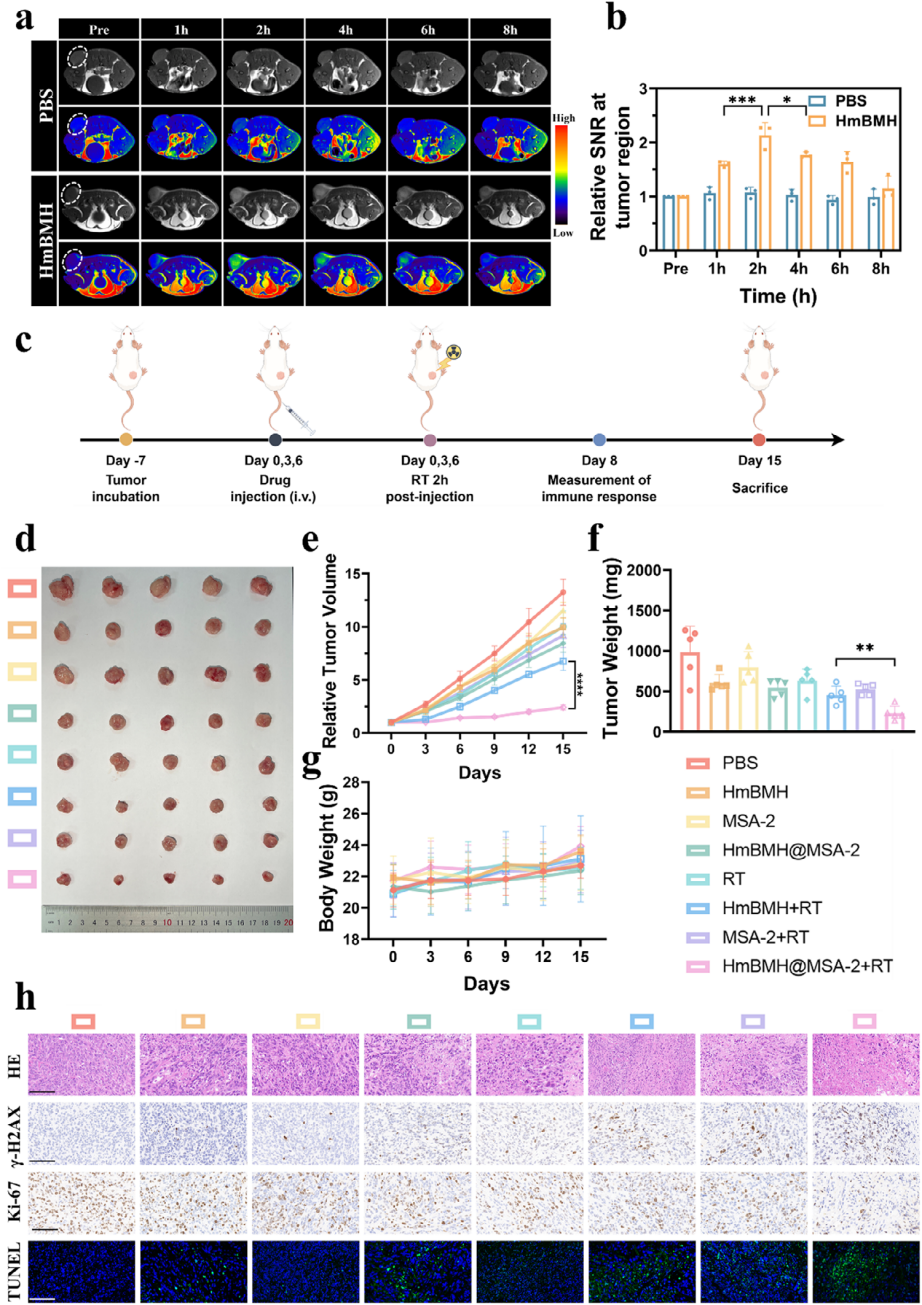
In vivo MR imaging and antitumor effect. a) The T1‐weighted MR and corresponding pseudo‐color images and b) the calculated relative SNR at tumor region of 4T1 tumor‐bearing mice before and after intravenous injection of PBS or HmBMH. c) Schematic illustration of the antitumor experiment. d) Photograph of tumors collected from sacrificed mice after different treatments. e) Relative tumor volume of mice from different groups. f) Tumor weights of sacrificed mice after different treatments. g) Body weight curves of mice with different treatments. h) H&E, 𝛾‐H2AX, Ki‐67, and TUNEL staining of the tumors extracted from mice after various treatments, scale bar: 100 µm. Data were given as mean ± S.D. (n ≥ 3). Statistical significance was calculated via one‐way ANOVA with Tukey's test: **p < 0.01, ****p < 0.0001.

Since HmBMH has shown excellent potential as an MRI contrast agent in vitro, we explored its MRI enhancement effects in vivo. Prior to conducting the in vivo experiments, a hemolysis experiment was performed to assess biocompatibility. Even at a concentration of 1000 ug mL^−1^, HmBMH exhibited a hemolysis rate of less than 5% (Figure S16, Supporting Information). We also conducted in vivo toxicity experiments of the HmBMH, including acute (1 d) and chronic (14 d and 28 d) toxicity studies. We measured key biochemical indicators in the blood (alanine aminotransferase, aspartate aminotransferase, urea, and creatinine), and the results showed that all values were within the normal range for both PBS and HmBMH groups, with no significant differences between them (Figure S17, Supporting Information). These results indicate that the nanomaterials had good biocompatibility and could be used for subsequent in vivo studies. Pre‐ and post‐injection T1‐weighted MR images were acquired from 4T1 tumor‐bearing BALB/c mice at various time points to validate the contrast effects of HmBMH. The MR images and the corresponding quantitative analysis showed that the T1 signal intensity at the tumor site gradually increased over time (**Figure** **5**a). The signal‐to‐noise ratio (SNR) in the tumor region peaked at 2 h post‐injection (Figure 5b). These results indicate that HmBMH accumulated at the tumor site and, upon GSH stimulation, decomposed to release Mn ions, enabling TME‐activatable self‐enhanced tumor diagnosis. Our experimental results suggest that 2 h post injection of HmBMH provided a suitable therapeutic time window for subsequent in vivo radiation therapy.

Encouraged by the excellent results of the in vitro experiments, we examined the antitumor effects of HmBMH@MSA‐2 in vivo (Figure 5c). As depicted in Figure 5d–f, tumors in the HmBMH@MSA‐2 group exhibited mild suppression compared to the PBS group, indicating the potential of HmBMH@MSA‐2 nanosystems in cancer treatment through CDT and TME‐responsive drug release. The tumors were significantly suppressed in mice receiving the HmBMH@MSA‐2 + RT combination treatment, confirming the ability of these multifunctional drug‐carrying nanosystems to sensitize RT and enhance the effect of immunotherapy. Notably, there was no significant decrease in body weight of the mice during the treatment period (Figure 5g), indicating the absence of significant adverse effects associated with the treatment. To further assess biocompatibility, histological analysis was conducted on the major organs (heart, liver, spleen, lungs, and kidneys) of experimental animals, and serum biochemical markers, including serum alanine aminotransferase (ALT), aspartate aminotransferase (AST), urea, and creatinine, were evaluated. Histological examination revealed no changes in the major organs (Figure S18, Supporting Information) and serum biochemical markers showed no significant differences between the groups (Figure S19, Supporting Information), indicating the biosafety of the treatment regimen.

In addition, the Hif‐1α immunohistochemical staining experiment was performed. As depicted in Figure S20, Supporting Information, the HmBMH group exhibited a significant decrease in Hif‐1α expression in the tumor region compared to the PBS group. These findings indicate that HmBMH played a crucial role in alleviating hypoxia in the TME. Additionally, tumor necrosis, DNA damage, cell proliferation and apoptosis were assessed using H&E, γ‐H2AX antigen, Ki‐67, and TUNEL staining. The results of H&E and γ‐H2AX staining revealed increased levels of tumor necrosis and DNA damage in the HmBMH@MSA‐2 + RT group (Figure 5h). Furthermore, the HmBMH@MSA‐2 + RT group exhibited a reduced Ki‐67‐positive proliferation rate and an elevated TUNEL‐positive apoptosis rate, suggesting potent antitumor efficacy via inhibition of tumor cell proliferation (Figure 5h). Collectively, our in vivo experimental results underscore the advantages of HmBMH@MSA‐2 combined with radiotherapy as an antitumor therapy.

### 2.6 In Vivo Immune Response Elicited by HmBMH@MSA‐2 Plus X‐ray

**Figure 6:**
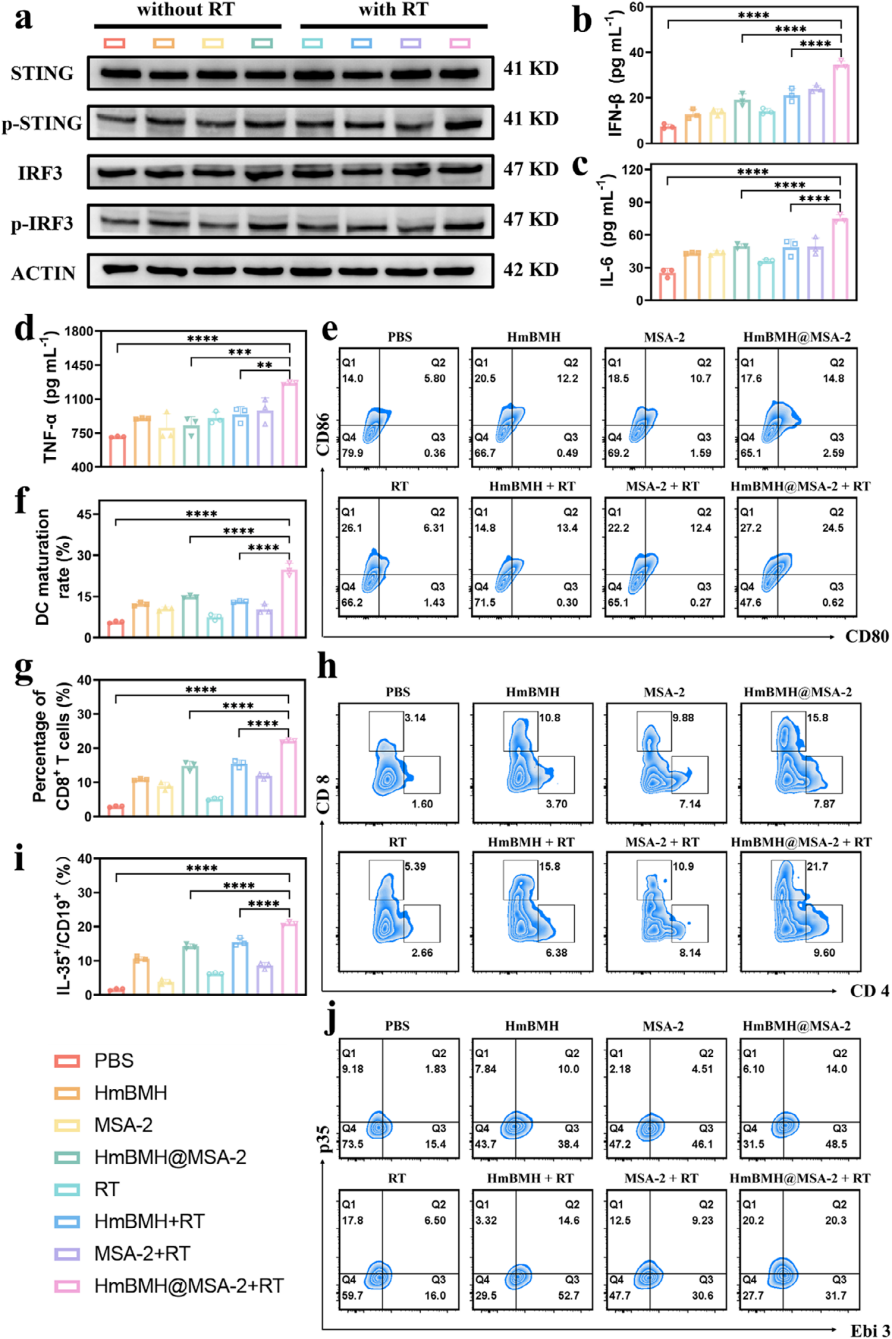
In vivo immune response elicited by HmBMH@MSA‐2 plus X‐ray. a) Western blot analysis for proteins related to the cGAS/STING signaling pathway. b–d) ELISA results of proinflammatory cytokine (IFN‐β, IL‐6, and TNF‐𝛼) levels in mice serum. e,f) Flow cytometry analysis of DCs in tumor tissues from mice with different treatments and the corresponding quantification. g,h) Flow cytometry analysis of CD4+T cells and CD8+T cells in tumor tissues from mice with different treatments and the corresponding quantification. i,j) Flow cytometry analysis of IL‐35‐producing Breg cells in tumor tissues from mice with different treatments and the corresponding quantification. Data were given as mean ± S.D. (n = 3). Statistical significance was calculated via one‐way ANOVA with Tukey's test: ****p < 0.0001.

Motivated by the results of the tumor suppression experiments, we further evaluated the nanosystem‐mediated immune responses in vivo. In line with the in vitro findings, the expression levels of key phosphorylated proteins (p‐STING and p‐IRF3) were significantly elevated in the HmBMH@MSA‐2 + RT group (**Figures** **6**a, and S21, Supporting Information). Given that activation of the STING pathway stimulates the release of IFN‐β and pro‐inflammatory cytokines, we further assessed mice serum cytokines by ELISA. In the HmBMH@MSA‐2 + RT group, the secretion of IFN‐ 𝛽, IL‐6, and tumor necrosis factor alpha (TNF‐α) in mice serum increased (Figure 6b–d). These findings validated the potential of the nanosystems to enhance the STING pathway.

Given the established role of IFN‐β in activating innate immunity,^[^ ^43^ ^]^ we investigated whether the nanosystems could promote DC maturation in vivo. Flow cytometry analysis revealed that the percentage of mature DCs in the HmBMH@MSA‐2+RT group (24.5%) was ≈1.7‐ and 4.2‐fold higher than those in the HmBMH@MSA‐2 (14.8%) and control groups (5.8%), respectively (Figure 6e,f). Subsequently, we focused on CTLs in tumors given their pivotal role as antitumor defenders and as an important weapon for killing tumors. The CD8^+^ CTL and CD4^+^ helper T cell populations in the tumors were also assessed using flow cytometry. As anticipated, the HmBMH@MSA‐2 + RT group exhibited a significantly higher frequency of CD8^+^ and CD4^+^ T cells in tumors than the control group (Figure 6g,h). Our results suggest that STING activation can effectively induce DC maturation and T cell activation.

Simultaneously, we noted an increase in the population of IL‐35^+^ Breg cells in the tumors. IL‐35, a heterodimer consisting of p35 and EBi3 subunits, belongs to the IL‐12 cytokines family.^[^ ^44^ ^]^ IL‐35 plays a role in the interplay between malignant tumor cells and surrounding immune cells within the TME, fostering an immunosuppressive environment and hindering effective anti‐tumor immune responses. Flow cytometry analysis revealed that the proportion of IL35^+^ Breg cells (CD19^+^IL‐35^+^) within the tumors of the HmBMH@MSA‐2+RT group (20.3%) was ≈4.5‐ and 11.1‐fold higher than those in the MSA‐2 (4.51%) and control groups (1.83%), respectively (Figure 6i,j). Recent studies have found that resistance to STING monotherapy in solid tumors is associated with an unexpected surge in the number of Breg cells that produce IL‐35, leading to a decrease in the density of NK cells within the tumor.^[^ ^15^, ^17^ ^]^ Consequently, a strategic combination with anti‐IL‐35 blockade is anticipated to augment tumor immunotherapy and reshape the tumor immune microenvironment.

### 2.7 Combined Anti‐IL‐35 Blockade Promoted NK Cells Response to Enhance Innate Immunity

STING activation is known to activate DCs, leading to tumor‐specific CD8^+^ T cell activation and stimulation CD8^+^ T cells to produce increased levels of IFN‐l.^[^ ^18^, ^45^ ^]^ In contrast to the antitumor effects of STING agonists on T cells, Li et al. showed that STING activation induces protumorigenic effects by increasing the number of IL‐35‐producing Breg cells in tumors.^[^ ^15^ ^]^ Increased IL‐35 expression reduces the density of NK cells in tumors, resulting in decreased granzyme B production and cytotoxicity. NK cells are essential components of the innate immune system. They traverse blood vessels to perform immune surveillance and promptly detect and activate immune defenses to rapidly kill diseased and cancerous cells, demonstrating their role as the first line of defense against cancer.^[^ ^46^, ^47^ ^]^ Therefore, restoring the number and function of NK cells is critical for tumor intervention.

**Figure 7:**
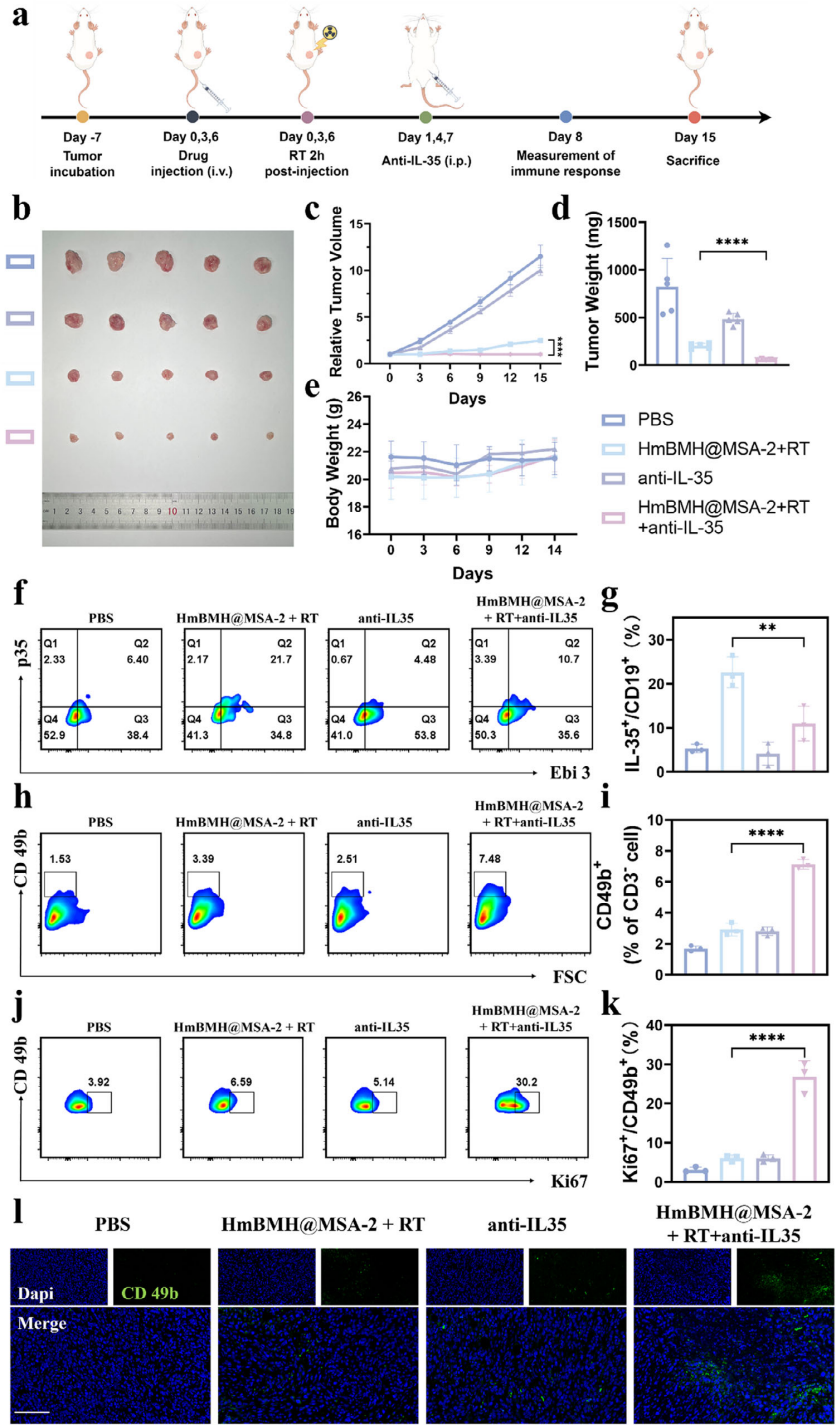
Combined anti‐IL‐35 blockade promoted NK cells response. a) Schematic illustration of the antitumor experiment in combination with anti‐IL‐35. b) Photograph of tumors collected from sacrificed mice after different treatments. c) Relative tumor volume of mice from different groups. d) Tumor weights of sacrificed mice after different treatments. e) Body weight curves of mice with different treatments. f,g) Flow cytometry analysis of IL‐35‐producing Breg cells in tumor tissues from mice with different treatments and the corresponding quantification. h,i) Flow cytometry analysis of NK cells in tumor tissues from mice with different treatments and the corresponding quantification. j,k) Flow cytometry analysis of Ki67‐expressing NK cells in tumor tissues from mice with different treatments and the corresponding quantification. l) Immunofluorescence images of tumor sections for NK cells stained with CD49b (green) and nuclear (blue), scale bars: 100 µm. Data were given as mean ± S.D. (n ≥ 3). Statistical significance was calculated via one‐way ANOVA with Tukey's test: *p < 0.05, **p < 0.01, ****p < 0.0001.

To evaluate the therapeutic effect of HmBMH@MSA‐2+RT combined with the anti‐IL‐35 blockade in vivo (**Figure** **7**a), a specific neutralizing antibody against IL12 p35 (one subunit of IL35) was used to deplete IL35 in mice.^[^ ^48^ ^]^ As illustrated in Figure 7b–d, the combination of HmBMH@MSA‐2+RT and anti‐IL‐35 effectively suppressed tumor growth. Compared with the HmBMH@MSA‐2+RT group, the tumor volume significantly decreased following the addition of anti‐IL‐35 treatment. Conversely, treatment with anti‐IL‐35 alone modestly inhibited tumor growth. During the treatment period, no significant weight loss was observed in the mice (Figure 7e), and no histological changes were observed in the major organs (heart, liver, spleen, lungs, and kidneys) (Figure S22, Supporting Information). Serum biochemical markers such as ALT, AST, urea, and creatinine were not significantly different (Figure S23, Supporting Information). These findings suggest that combined anti‐IL‐35 blockade therapy safely and effectively inhibited tumor growth. Furthermore, therapeutic efficacy was evaluated by H&E, γ‐H2AX antigen, Ki‐67, and TUNEL staining (Figure S24, Supporting Information). Compared with the HmBMH@MSA‐2+RT group, the HmBMH@MSA‐2+RT combined with anti‐IL‐35 group exhibited increased levels of tumor necrosis and DNA damage, with effective inhibition of tumor cell proliferation. Overall, the combination of HmBMH@MSA‐2 with RT and anti‐IL‐35 synergistically inhibited tumor growth.

Given the remarkable tumor‐suppressing effects of the combination therapy, we further investigated the anti‐tumor immune‐enhancing effects induced by HmBMH@MSA‐2 plus RT combined with anti‐IL‐35. As shown in Figure 7f,g, the combined IL‐35 blockade significantly decreased the infiltration of CD19^+^IL‐35^+^ cells in mouse tumors, compared to the HmBMH@MSA‐2+RT group. Motivated by this finding, we explored the infiltration of NK cells into mouse tumors. As depicted in Figure 7h,i, there was a slight increase in the number of NK cells that infiltrated mouse tumors in both the anti‐IL‐35 and HmBMH@MSA‐2+RT groups. Notably, the HmBMH@MSA‐2+RT+anti‐IL‐35 group exhibited a significant 2.20‐fold increase in NK cell infiltration compared to the HmBMH@MSA‐2+RT group. Additionally, the frequency of Ki67‐positive NK cells was 4.58‐fold higher in the HmBMH@MSA‐2+RT+anti‐IL‐35 group than in the HmBMH@MSA‐2+RT group (Figure 7j,k). We also assessed the infiltration of NK cells in the tumor at different time points of 24 h, 48 h, and 72 h after treatment. As shown in Figure S25, Supporting Information, the infiltration of NK cells in the tumors of mice treated with combined anti‐IL‐35 therapy was significantly higher than that in the HmBMH@MSA‐2 + RT group at 24 h, 48 h, and 72 h. By analyzing the changes in the relative number of tumor – infiltrating NK cells at different time points, it was found that the combined anti‐IL‐35 therapy group showed an increase compared to the PBS group and remained stable between 24 and 72 h (Figure S26, Supporting Information). These results suggest that the combination of anti‐IL‐35 therapy effectively sustained the immune response and significantly boosted NK cell antitumor effects.

NK cells secrete IFN‐γ, granzyme B, and perforin, which enhance tumor immunogenicity, thereby activating the immune response and inducing tumor cell apoptosis.^[^ ^49^, ^50^ ^]^ Following the HmBMH@MSA‐2+RT+anti‐IL‐35 treatment, the levels of IFN‐γ, granzyme B, and perforin in serum were significantly elevated (Figure S27, Supporting Information). Furthermore, we assessed the infiltration of NK cells (denoted as CD49b‐positive cells) into tumors using immunofluorescence analysis. The HmBMH@MSA‐2+RT+anti‐IL‐35 group exhibited significantly increased green fluorescence of CD49b‐positive cells (Figure 7l), indicating enhanced NK cell infiltration in the tumor. Overall, the combination of HmBMH@MSA‐2 and RT with the anti‐IL‐35 neutralizing antibody facilitated NK cell infiltration and cytotoxicity in tumors, effectively enhancing innate immunity.

In addition, we have conducted additional experiments using a distant tumor model to simulate metastasis (Figure S28a, Supporting Information). 4T1 tumor‐bearing BALB/c mice were subjected to distant tumor inoculation following different therapies after being randomly assigned to four treatment groups, with the growth of distant tumors and immune effects being evaluated. As shown in Figure S28b,c, Supporting Information, the HmBMH@MSA‐2+RT combined with anti‐IL‐35 group effectively suppressed distant tumor growth, suggesting that the combined anti‐IL‐35 therapy has the potential to inhibit tumor metastasis. Meanwhile, no obvious weight loss occurred in the mice during this period, indicating that the combined anti‐IL‐35 therapy can suppress distant tumor growth safely and effectively (Figure S28d, Supporting Information). Given the significant inhibitory impact of the combination therapy on distant tumors, we delved into the immune effects within the distant tumors. As depicted in Figure S28e,f, Supporting Information, compared to the HmBMH@MSA‐2+RT group, a significant increase in NK cells infiltrating mice tumors was observed in the HmBMH@MSA‐2+RT+anti‐IL‐35 group. Moreover, compared to the HmBMH@MSA‐2+RT group, the HmBMH@MSA‐2+RT combined with anti‐IL‐35 group showed a substantial rise in Ki67‐positive NK cells (Figure S28g,h, Supporting Information). These findings indicated that the combination of anti‐IL‐35 therapy has potential to trigger an immune response to combat tumor metastasis in vivo.

## 3 Conclusion

Herein, we successfully constructed a TME‐responsive hollow mesoporous shell‐like nanorod‐based nanosystem, HmBMH@MSA‐2, for the real‐time imaging and enhancement of radioimmunotherapy. Upon exposure to GSH, the nanosystem underwent responsive degradation, releasing the small‐molecule STING agonist MSA‐2 and Mn ions. This system simultaneously induced mitoDNA and nDNA damage through a combination of CDT and RT. The accumulated dsDNA fragments escaped from cancer cells, thereby activating the cGAS‐STING signaling pathway and triggering a robust immune response. This significantly promoted the activation of DCs, leading to increased infiltration of CTLs into the tumor site. However, we also noted an increase in the number of IL‐35^+^ Breg cells within the tumor, potentially compromising the efficacy of tumor treatment. Given the STING agonist‐mediated crosstalk between Breg cells and NK cells, anti‐IL‐35 blockade therapy was introduced as a combination treatment. The results demonstrated that this strategy effectively decreased the number of IL‐35‐producing Breg cells, enhanced NK cell infiltration into the tumor, and potentiated innate immunity, further boosting the efficacy of immunotherapy. Finally, the TME‐specific release of Mn ions enabled activatable T1‐weighted MR imaging. Overall, the HmBMH@MSA‐2 nanosystem, in conjunction with anti‐IL‐35 blockade therapy, offers a promising theranostic strategy for reversing the tumor‐induced immunosuppressive microenvironment, enhancing the activation of innate immunity, and improving cancer radioimmunotherapy efficacy. However, the clinical translation may face challenges related to large‐scale production and long‐term safety, which require further investigation.

## 4 Experimental Section

### Materials

Bi (NO_3_)_3_⋅5H_2_O, Na_2_S⋅9H_2_O, hyaluronic acid (HA), and MSA‐2 were purchased from Macklin, Shanghai, China. Polyvinylpyrrolidone was obtained from Solarbio, Beijing, China. Potassium permanganate (KMnO_4_) was purchased from Chemical Reagent Factory, Guangzhou, China. The CCK‐8 assay and reactive oxygen species assay (DCFH‐DA) were from Beyotime Biotechnology, Shanghai, China. RPMI‐1640 medium, Dulbecco's Modified Eagle Medium (DMEM), fetal bovine serum (FBS), Penicillin‐Streptomycin, and 0.05% trypsin‐EDTA were purchased from Gibco, Thermo Fisher, USA.

### Cell Lines and Animals

The mouse breast cancer cell line 4T1 and the MC3T3‐E1 cells were donated by the Research Center of Clinical Medicine, Nanfang Hospital (Guangzhou, China). The 4T1 cells were cultured in RPMI‐1640 medium, while MC3T3‐E1 cells were seeded in DMEM containing 10% FBS, 100 units mL^−1^ of penicillin and 100 µg mL^−1^ of streptomycin. Cells were incubated in a humidified atmosphere of 5% CO_2_, 1% O_2_, and 94% N_2_ at 37 °C. Female Balb/c mice (4–6 weeks) were purchased from Southern Medical University Laboratory Animal Center (Guangzhou, China). All animal experimental procedures were approved by the Experimental Animal Ethics Committee of Southern Medical University.

### Synthesis of HmBM and HmBMH

Synthesis of Bi_2_S_3_ NRs was modified based on prior literature.^[^ ^25^ ^]^ After the as‐prepared Bi_2_S_3_ NRs were dispersed in ultrapure water (0.5 mg mL^−1^) under vigorous stirring, a solution of KMnO_4_ (60 mg) was slowly added and reacted at room temperature. Then, the products were collected and washed to obtain the HmBM NPs. For hyaluronic acid (HA) coating, 5 mg of HmBM was dissolved in 10 mL of ultrapure water. Subsequently,15 mg of HA was added into the HmBM solution, followed by stirring for 12 h to obtain HmBMH.

### Synthesis of HmBM@MSA‐2 and HmBMH@MSA‐2

For MSA‐2 loading, HmBM and different concentrations of MSA‐2 in DMSO were mixed under magnetic stirring for 24 h. Centrifugation (12 000 rpm, 15 min) was used to extract free MSA‐2, which was then washed with ultrapure water obtain HmBM@MSA‐2. The amount of MSA‐2 was assessed by measuring the UV–vis absorption peak at 322 nm after centrifugation. For HA coating, the method was the same as mentioned above.

### Characterization

Transmission electron microscopy (TEM) images and high‐angle annular dark‐field (HADDF)‐scanning transmission electron microscopy (STEM)‐based elemental mapping images were required by a JEOL JEM‐2100F TEM. X‐ray photoelectron spectroscopy (XPS) spectra was determined by an ESCALAB Xi+ instrument, to analyze the valence of the Mn, O, C, and Bi component of HmBM NPs. The pore size distribution of the samples was determined using the Brunauer‐Emmet‐Teller (BET) method. The UV–vis absorption spectra were recorded on an UV‐2600 UV–vis spectrophotometer from Shimadzu, Japan. The zeta potential of samples was examined by a Zetasizer Nano ZS (Malvern). The electron‐spin‐resonance (ESR) spectra were determined using a Bruker A300/E500 spectrometer. The Fourier transform infrared (FT‐IR) spectrum was acquired using a Nicolet iS50 FT‐IR spectrometer.

### The Degradation of HmBM and Drug Release Study

A standard method was employed to assess the GSH dependent degradation ability by measuring the accumulated degradation of Mn via ICP‐OES. To study the release profile of MSA‐2, a specific quantity of HmBM@MSA‐2 was dialyzed in various buffer solutions (0, 1, and 10 mM GSH) at room temperature. UV–vis spectrophotometry was utilized to quantify the amount of MSA‐2 released at various time points.

### GSH Depletion Assessment

GSH (1 mM) was added into pure water and varying concentrations of HmBM NPs solution. Following incubation at 37 °C for 1 h, the GSH content in each solution was measured using the Reduced Glutathione (GSH) Content Assay Kit (Solarbio, Beijing, China).

### Detection of ROS

First, 25 mM NaHCO_3_/5% CO_2_ buffer solution containing HmBM (0.5 mM of Mn) and varying concentrations of GSH (0, 1, and 10 mM) was shaken for 1 h at 37 °C and the supernatant was collected by centrifugation. Subsequently, the necessary quantities of MB solution and H_2_O_2_ solution were added to achieve a final mixture containing 10 µg mL^−1^ MB and 8 mM H_2_O_2_. The mixture was then placed in a 37 °C thermostatic bath for 30 min. The absorbance change (660 nm) of MB was then measured.

### Real‐Time O_2_ Generation

HmBM (200 µM of Mn) and 1 mM H_2_O_2_ was injected into a sealed container containing 15 mL PBS solution under constant stirring and coupled with the oxygen probe (ST300D portable Dissolved Oxygen Meter, OHAUS, USA), then the concentration of O_2_ was monitored in real time. PBS solutions with or without the addition of 1 mM H_2_O_2_ served as controls.

### In Vitro MR Imaging and GSH‐Mediated Activatable MR Imaging

Aqueous dispersions of HmBM at various concentrations (0, 0.025, 0.05, 0.10, 0.20, and 0.40 mM Mn) were scanned by a 3.0 T clinical MRI scanner (Philips Ingenia). For GSH‐mediated activatable MR imaging, the HmBM aqueous solutions with the same concentrations treated with GSH (0, 1 mM) were also scanned.

### In Vitro Cell Cytotoxicity Assay

The 4T1 cells (8000 cells well^−1^) and MC3T3‐E1 cells (8000 cells well^−1^) were seeded in a 96‐well plate. After 24 h of incubation, the medium was replaced with HmBMH, dispersed in medium with various concentrations and incubated for another 24 h. Subsequently, cell viability was evaluated by the CCK‐8 assay.

### Western Blot Assay

Protein lysate was prepared from tumor tissues or cells for protein extraction. The proteins were transferred to polyvinylidene difluoride membranes and immunoblotting was performed using antibodies. Protein levels were then analyzed using the Image Lab system.

### Cellular Uptake Ability of HmBMH

To assess the specific uptake of HmBMH by 4T1 cells through HA receptors, 4T1 and MC3T3‐E1 cells were seeded in confocal dish and cultured for 24 h. Subsequently, the cells were incubated with RhB‐conjugated HmBMH for 3 h. Following incubation, the treated cells were fixed, stained with DAPI, and visualized using CLSM. To further confirm the specificity of HA binding to CD44, an excess amount of free HA (2 mg mL^−1^) was added and pre‐incubated with 4T1 cells. Afterward, the 4T1 cells were co‐incubated with RhB‐conjugated HmBMH for another 4 h. The cells were then fixed with 4% paraformaldehyde, stained, and observed.

### Intracellular ROS Analysis

DCFH‐DA was used as a probe to detect the intracellular generation of ROS. The cells were seeded in a 96‐well plate for 24 h. Following cell attachment, HmBMH was added and incubated for 6 h. After washing with PBS buffer, DCFH‐DA was added into each well for 30 min in the dark. Subsequently, fluorescence images were captured by a fluorescent microscope (Nikon Eclipse T1‐U, Tokyo, Japan). ROS levels were further analyzed by flow cytometry (BD LSRFortessaTM Cell Analyzer, USA).

### Evaluation of Mitochondrial Function

4T1 cells were pre‐treated with HmBMH for 12 h. Subsequently, we evaluated the mitochondrial membrane potential using the JC‐1 Assay Kit (Beyotime, Shanghai, China) via flow cytometry. Furthermore, we determined the intracellular mitochondrial ROS (mitoROS) levels using the mitoSOX fluorescence (red) probe (Yeasen, Shanghai, China). Last, the changes in mitochondrial permeability transition pore (mPTP) opening were detected by mPTP kit (Beyotime, Shanghai, China), following to the manufacturers’ instructions.

### Immunofluorescence Analysis of 8‐OH‐dG

First, the 4T1 cells were collected and seeded onto a confocal dish. After attachment, the cells were treated with HmBMH. Subsequently, the cells were fixed with 4% paraformaldehyde for 15 min, and permeabilized with 0.3% Triton X‐100 solutions for 15 min. The cells were then incubated with both 8‐OHdG antibody and TOMM20 antibody. 12 h later, the cells were incubated with secondary antibody for 1 h, and with DAPI for 5 min. Ultimately, the mitochondrial and Oxidized mitochondrial DNA (Ox‐mitoDNA) were assessed by immunofluorescence.

### Intracellular Clonogenic Assay and Immunofluorescence Analysis of γ‐H2AX and PicoGreen

4T1 cells were seeded in a 6‐well plate and incubated for 24 h, and treated with PBS and HmBMH, respectively. Following incubation for 6 h, cells were exposed to 0, 2, 4, and 6 Gy X‐ray radiation (MultiRad Faxitron, USA), respectively. The cells were then incubated for another 7 days. Last, the cells were fixed with paraformaldehyde and stained with crystal violet. The survival fraction was calculated to evaluate the impact of various treatment on the cells.

For immunofluorescence analysis of γ‐H2AX and PicoGreen, 4T1 cells were seeded in CLSM dishes and cultured for 24 h. The cells were then treated as mentioned above. Following incubation for 24 h, the cells were stained using the DNA damage Assay Kit by γ‐H2AX Immunofluorescence (Beyotime, China), Picogreen dsDNA Quantitation Reagent (Yeasan, China) and DAPI, and finally observed and captured by fluorescence microscope.

### ELISA Analysis of IFN‐β and IL‐6

4T1 cells were cultured in a 6‐well plate for 24 h, then divided into eight groups: PBS, HmBMH, MSA‐2, HmBMH@MSA‐2, RT, HmBMH+RT, MSA‐2+RT, and HmBMH@MSA‐2+RT. Following the various treatments, supernatants were collected after 24 h for IFN‐β and IL‐6 detection using Mice ELISA Kits (Meimian Industrial, Jiangsu, China).

### DCs Maturation In Vitro

To evaluate the immunogenic activity in vitro, the tumor cells supernatant was obtained using the previously described treatment method and incubated it with BMDCs for 24 h. Subsequently, the treated BMDCs were stained with anti‐mice anti‐CD11c, anti‐CD80, and anti‐CD86 antibodies (Invitrogen, California, USA). The maturation of DC cells was analyzed by flow cytometry.

### In Vivo MRI Assessment

4T1 tumor‐implanted mice (female, 4–6 weeks) were used for in vivo MR imaging. Mice were anesthetized and scanned using a MRI animal coil to obtain the coronal T1‐weighted images before and after intravenous injection of 200 µL of PBS or HmBMH ([Mn]: 4 mg kg^−1^). The parameters were set as follows: TR/TE = 450/15.4 ms, field of view = 40 mm × 40 mm, matrix = 200 × 170, slice thickness = 1 mm, number of excitations = 3. Regions of interest (ROIs) were manually drawn around the tumor of each mouse, and the corresponding pre‐ and post‐injection signal intensities were measured in the post‐processing workstation.

### Red Blood Cells Hemolysis In Vitro

Fresh erythrocyte suspensions were utilized to evaluate the biocompatibility of the HmBMH with blood in vitro. Briefly, the HmBMH NPs were dissolved in 1 mL PBS at various concentrations (6.25−1000 µg mL^−1^) and then mixed with 200 µL of erythrocytes suspension. The mixture was centrifuged (3000 rpm, 15 min) after incubation at 37 °C for 4 h. Subsequently, the UV–vis absorbance of the supernatant was measured to calculate the percentage of hemolysis. DI water and PBS were used as positive and negative controls, respectively.

### In Vivo Antitumor Effect

The 4T1 tumor‐bearing mice were randomly divided into 8 groups (n = 5 per group): (1) PBS, (2) HmBMH ([Mn]: 2 mg kg^−1^); (3) MSA‐2(2.7 mg kg^−1^); (4) HmBMH@MSA‐2 ([Mn]: 2 mg kg^−1^); (5) RT; (6) HmBMH+RT; (7) MSA‐2+RT; (8) HmBMH@MSA‐2+RT. X‐ray irradiation was performed 2 h after injection (2 Gy) every 3 days for a total of three irradiations. The volume (V) of tumor was measured at 3 days interval for 15 days using the formula as follows: V = length × width^2^/2. Relative tumor volumes (V/V0) were normalized to the initial volumes (V0). Additionally, the body weight and the tumor weight were recorded. After 15 days, all mice were sacrificed, and the blood samples of mice were collected for hematological and biochemical analysis. Last, H&E staining and the expression of TUNEL, γ H2AX, Ki‐67, and Hif‐1α expression in tumor tissues from each group were conducted to evaluate antitumor efficacy.

### In Vivo Immune Response Activation

To further assess the capacity of this combinational therapy to elicit an anti‐tumor immune response, the tumor tissue samples were collected and digested with RPMI‐1640 medium supplemented with DNase I (Solarbio, China) and Collagenase IV (Solarbio, China). Subsequently, the tumor tissues were filtered through cell strainers to obtain single‐cell suspensions. The cells were counted and stained with various antibody panels, following the manufacturer's protocols.

The collected cells were then incubated with anti‐CD11c, anti‐CD80, and anti‐CD86 antibodies to assess the abundance of mature DC cells in the tumors using flow cytometry. Additionally, the collected cells were stained with anti‐CD45, anti‐CD3, anti‐CD4, and anti‐CD8 antibodies (Invitrogen, California, USA) to quantify CD4^+^ or CD8^+^ T cells in the tumors. For intracellular cytokine staining, the cells were stimulated in vitro with RPMI‐1640 medium containing phorbol 12‐myristate 13‐acetate (PMA), ionomycin, Brefeldin A, and monensin, and then incubated at 37 °C for 4 h. Following this, the cells were incubated with anti‐CD45 and anti‐CD19 antibodies (Invitrogen, California, USA), fixed, permeabilized, and stained with anti‐EBI3 and anti‐p35 antibodies (R&D Systems, Minnesota, USA) to detect IL‐35‐producing Breg cells in tumors. The expression levels of TNF‐𝛼, IL‐6 and IFN‐𝛽 in mice serum were measured using ELISA kits (Meimian Industrial, Jiangsu, China) following the instructions.

### In Vivo Antitumor Effect Combined with Anti‐IL‐35

4T1 tumor‐bearing mice were randomly divided into 4 groups (n = 5 in each group): (1) PBS, (2) HmBMH@MSA‐2+RT ([Mn]: 2 mg kg^−1^); (3) anti‐IL‐35 (200 µg); (4) HmBMH@MSA‐2+RT+ anti‐IL‐35. X‐ray irradiation was administered 2 h after injection (2 Gy) every 3 days for a total of three irradiations. Anti‐IL‐35 (BioXCell, New Hampshire, USA) was administered via intraperitoneal injection every 3 days for three cycles. After the treatment, the volume (V) of tumor was measured at 3 days interval for 15 days using the formula as follows: V = length × width^2^/2. Relative tumor volumes (V/V0) were normalized to the initial volumes (V0). Moreover, the body weight and the tumor weight were recorded. After 15 days, all mice were sacrificed and the blood samples of mice were collected to evaluate the hematological and biochemical indexes. Last, the H&E staining and the expression of TUNEL, γ H2AX, and Ki‐67 of the tumors in each group were also performed to assess the antitumor efficacy.

### In Vivo Immune Response Activation Combined with anti‐IL‐35

The method for isolating single‐cell suspensions and identifying IL‐35‐producing Breg cells was as previously described. Furthermore, the collected cells were stained with anti‐CD45, anti‐CD3, anti‐CD49b, and anti‐Ki‐67 antibodies (Invitrogen, California, USA) to assess NK cell responses within the tumor. The expression levels of Granzyme b, perforin and IFN‐γ in mice serum were quantified using ELISA kits (Meimian Industrial, Jiangsu, China) following the instructions. The expression of CD49b on tumors in each group were also evaluated to assess NK cells activity.

### Ethics Approval and Consent to Participate

All animal care and in vivo studies were approved by the Animal Care and Use Committee of Southern Medical University and conducted in accordance with the ethical principles of the Ethics Committee for Animal Research (approval SMUL202402001).

### Statistical Analysis

Data are expressed as mean ± standard deviation (SD). All experiments were repeated at least three times. Statistical significance was determined using a Student *t* test for comparisons between two groups, and one‐way analysis of variance (ANOVA) followed by multiple comparisons with Tukey's test for multi‐group analyses within Prism 8.0 software (GraphPad Software). The *p* value < 0.05 was considered statistically significant (ns indicates *p* > 0.05; * *p* < 0.05; ** *p* < 0.01; *** *p* < 0.001; **** *p* < 0.0001).

## Conflict of Interest

The authors declare no conflict of interest.

## Supporting information



Supporting Information

## Data Availability

The data that support the findings of this study are available from the corresponding author upon reasonable request.
