# Supplementary material for: Nanosensitizer for Cancer Radioimmunotherapy via Anti‐IL‐35 Blockade Boosted Innate Immunity Activation
Source: Adv Sci (Weinh). 2025 Jun 26;12(36):e04252. doi: 10.1002/advs.202504252 (PMC12462979; doi:10.1002/advs.202504252)
Supplement: Supplementary file 1 — Supporting Information [file ADVS-12-e04252-s001.docx]

**Supporting Information**

**Nanosensitizer For Cancer Radioimmunotherapy via Anti-IL-35 Blockade Boosted Innate Immunity Activation**

*Yinfei Zheng^1,2,#^, Shuting Zheng^1,2,#^, Yushu Liao^2^, Zede Wu^1,2^, Chenxi He^1,2^, Qiuyu Li^2^, Honglei Hu^4^, Zheyu Shen^3^, Yikai Xu^1,*^, Chenggong Yan^1,*^, Bingxia Zhao^2,5,*^, Meirong Hou^1,*^*

^1^Department of Medical Imaging Center, Nanfang Hospital, Southern Medical University, Guangzhou 510515, China

^2^Cancer Research Institute, School of Basic Medical Sciences, Southern Medical University, Guangzhou 510515, China

^3^School of Biomedical Engineering, Southern Medical University, Guangzhou, Guangzhou 510515, China

^4^Department of Radiology, The Second Affiliated Hospital of Guangzhou Medical University, Guangzhou 510260, China

^5^ Experimental Education/Administration Center, School of Basic Medical Science, Southern Medical University, Guangzhou 510515, P. R. China

^#^ Yinfei Zheng and Shuting Zheng contributed equally to this work.

Corresponding Author:

Yikai Xu*: E-mail address: yikaixu917@gmail.com

Chenggong Yan*: E-mail address: ycgycg007@gmail.com

Bingxia Zhao*: E-mail address: bingxiaz@gmail.com

Meirong Hou*: E-mail address: houmr_fimmu@163.com


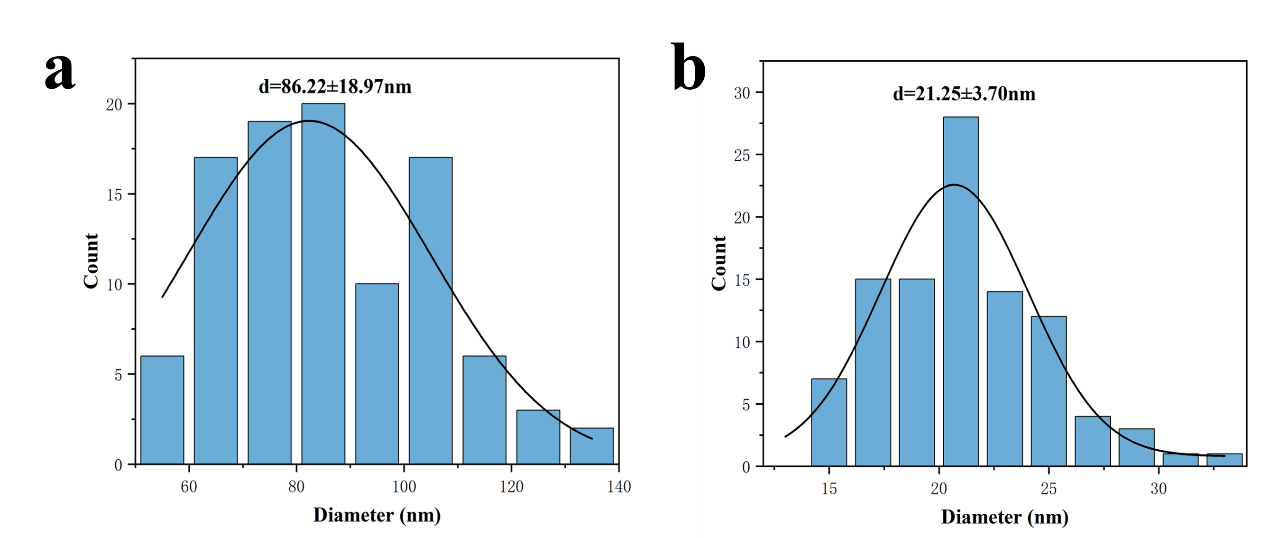


**Figure S1**. The average long diameter (a) and cross diameter (b) of HmBM NPs measured from the TEM images.


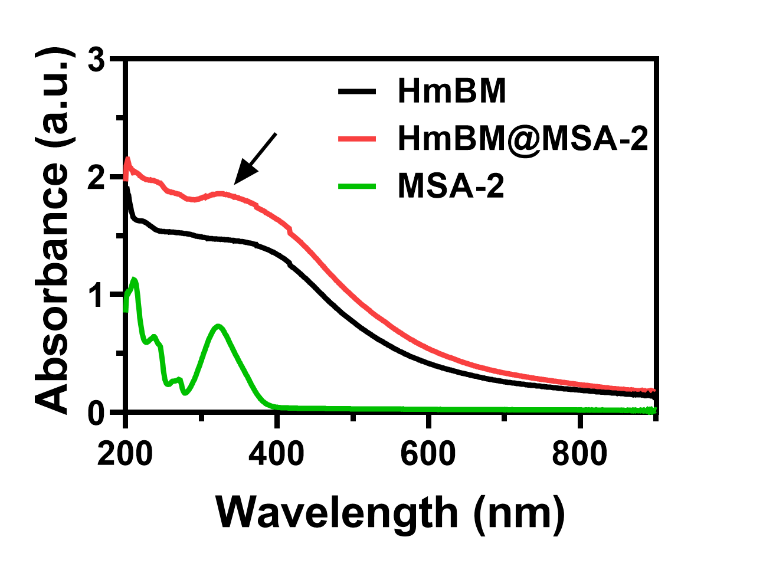


**Figure S2**. Spectrum of HmBM, MSA-2, and HmBM@ MSA-2 scanning using UV spectroscopy within the wavelength range of 200-900 nm.

**
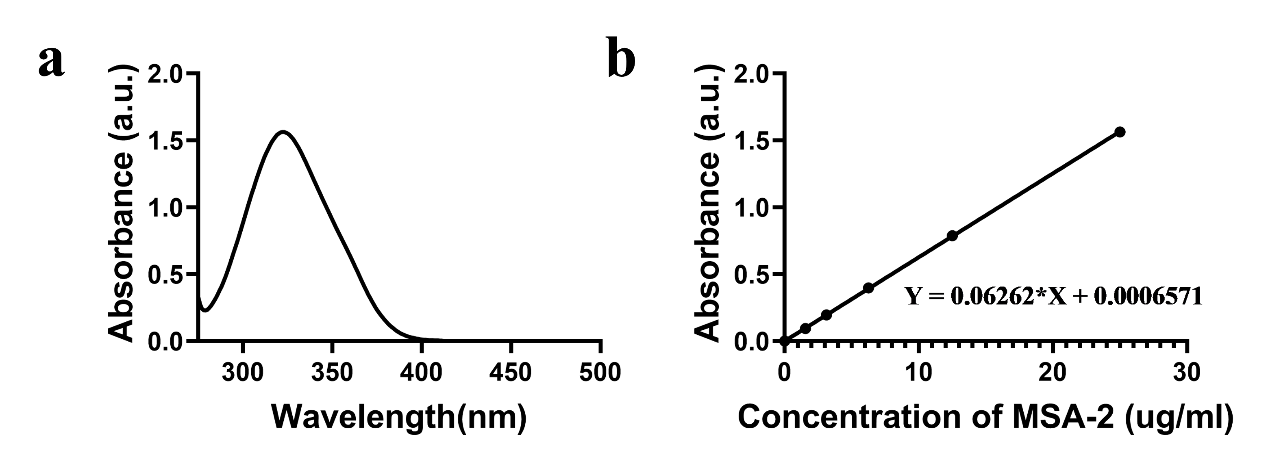
**

**Figure S3**. (a) UV spectrum analysis of MSA-2. (b) The linear relationship between absorbance (322 nm) and the concentration of MSA-2.

**
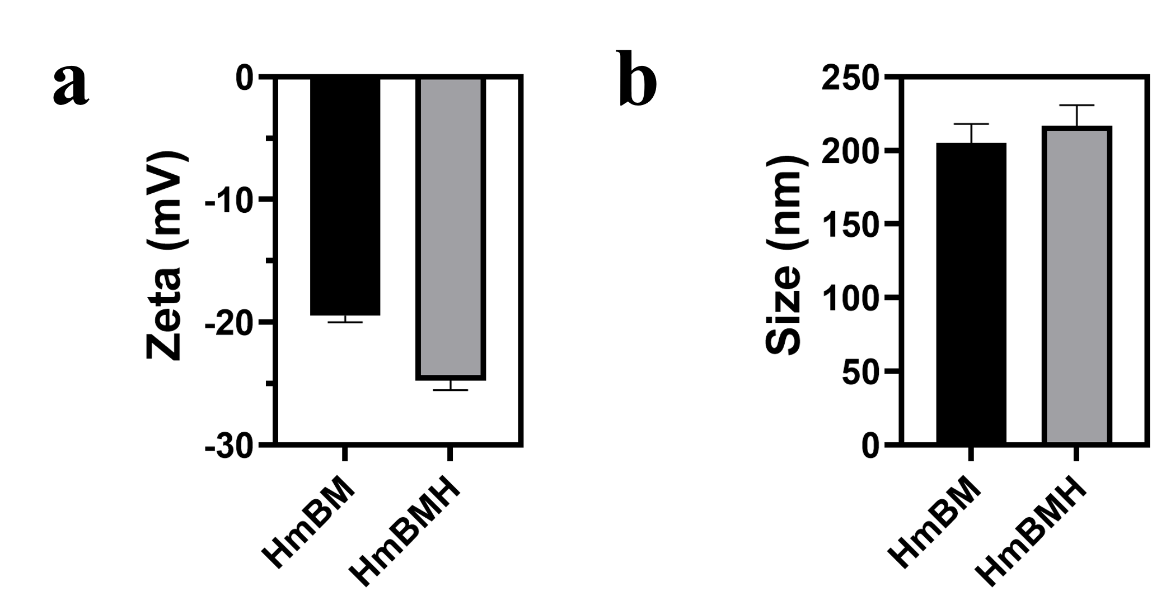
**

**Figure S4**. (a) The zeta potentials and (b) the particle sizes of HmBM and HmBMH. (n = 3)


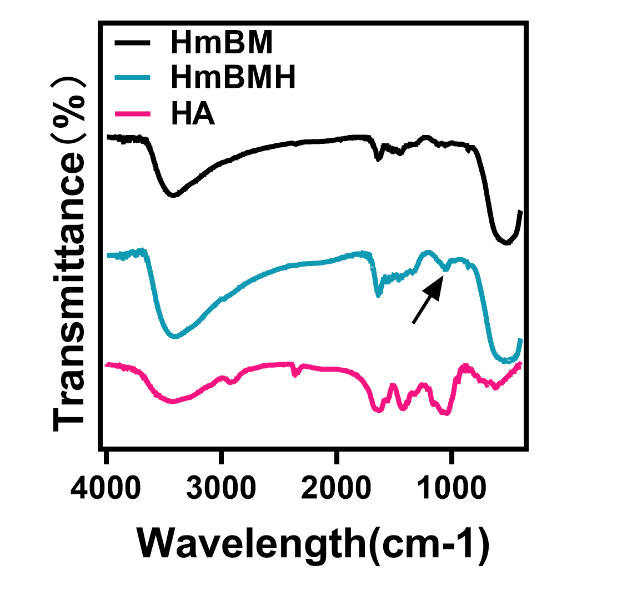


**Figure S5**. FTIR spectra of HmBM, HmBMH, and HA.


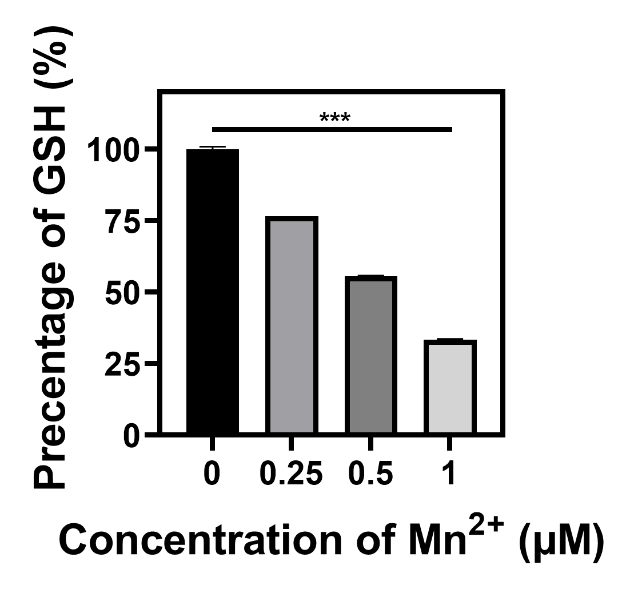


**Figure S6**. The relative concentrations of GSH in solutions containing various concentrations of HmBM. (n = 3)


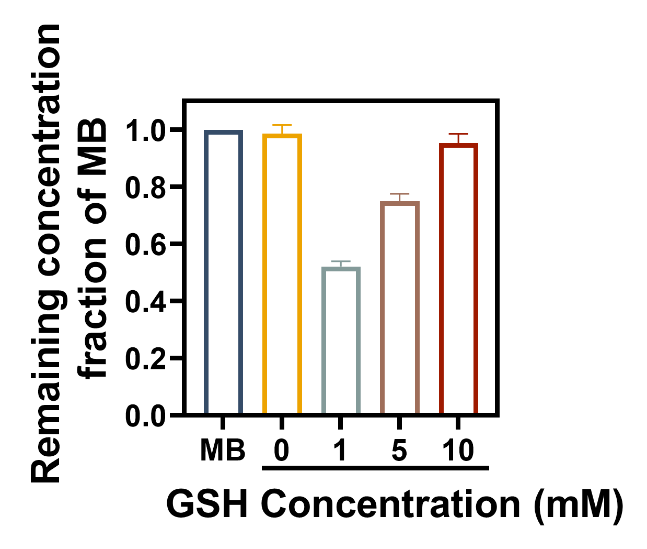


**Figure S7**. Relative quantification of MB at 660 nm. (n = 3)


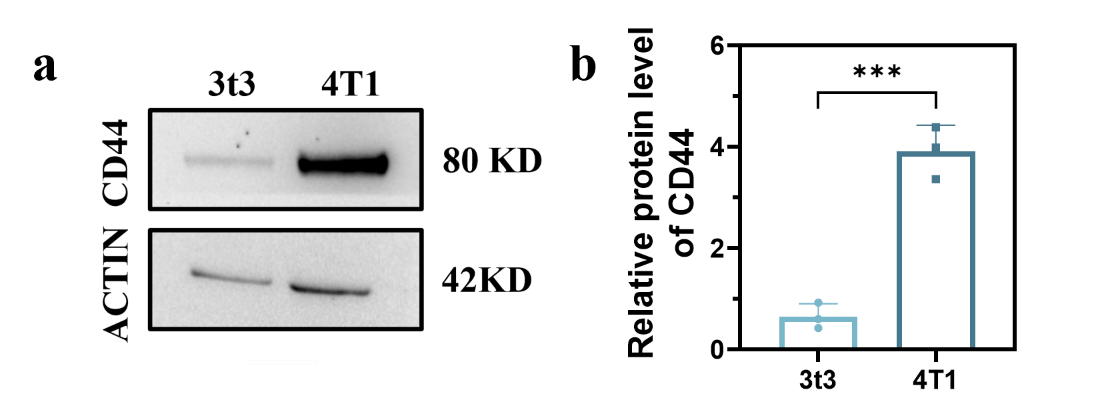


**Figure S8**. (a) Western blot analysis of CD44 expression level in 4T1 cells and 3t3 cells and (b) their relative quantification. (n = 3)


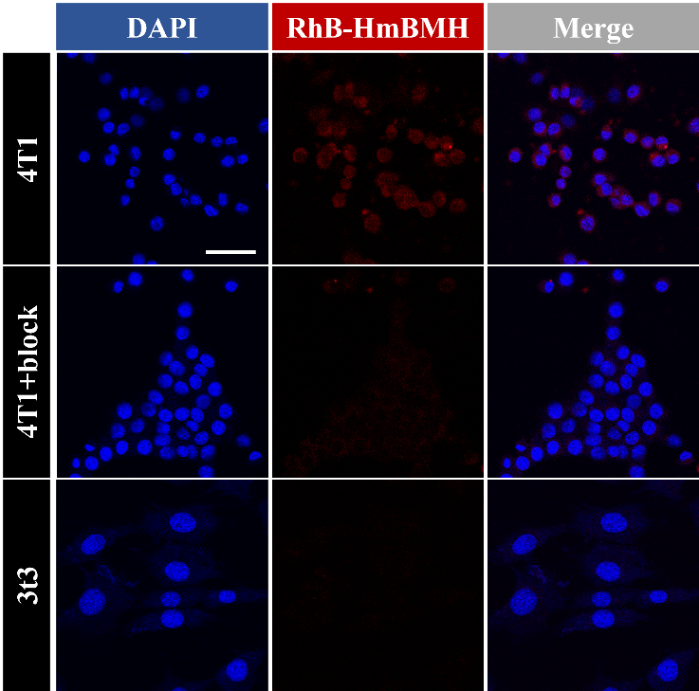


**Figure S9**. Cell uptake of RhB-HmBMH by 4T1 cells with or without excess HA and 3t3 cells observed in CLSM. Scale bar: 50 μm.


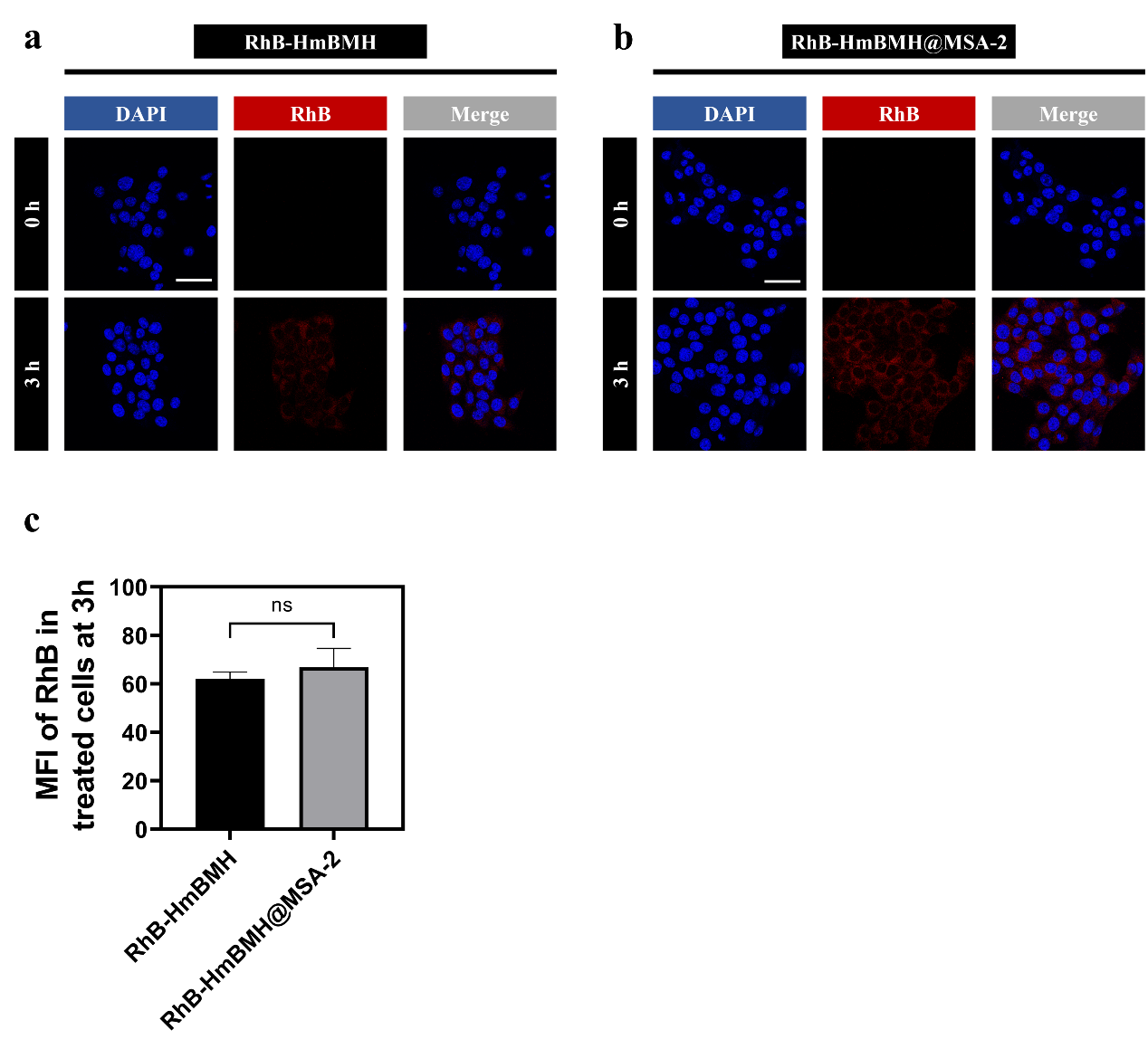


**Figure S10**. Cellular uptake of (a) RhB-HmBMH and (b) RhB-HmBMH@MSA-2 by 4T1 cells observed in CLSM. Scale bar: 50 μm. (c) Statistically analysis of intracellular RhB fluorescence intensity in 4T1 cells. (n = 3)


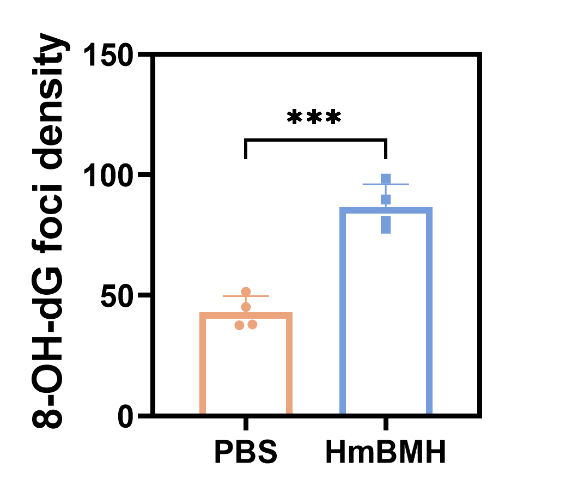


**Figure S11**. Quantitative analysis of 8-OH-dG foci density. (n = 4)


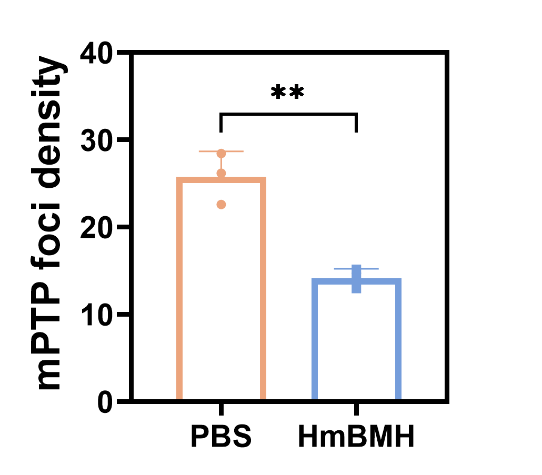


**Figure S12**. Quantitative analysis of mPTP foci density. (n = 3)


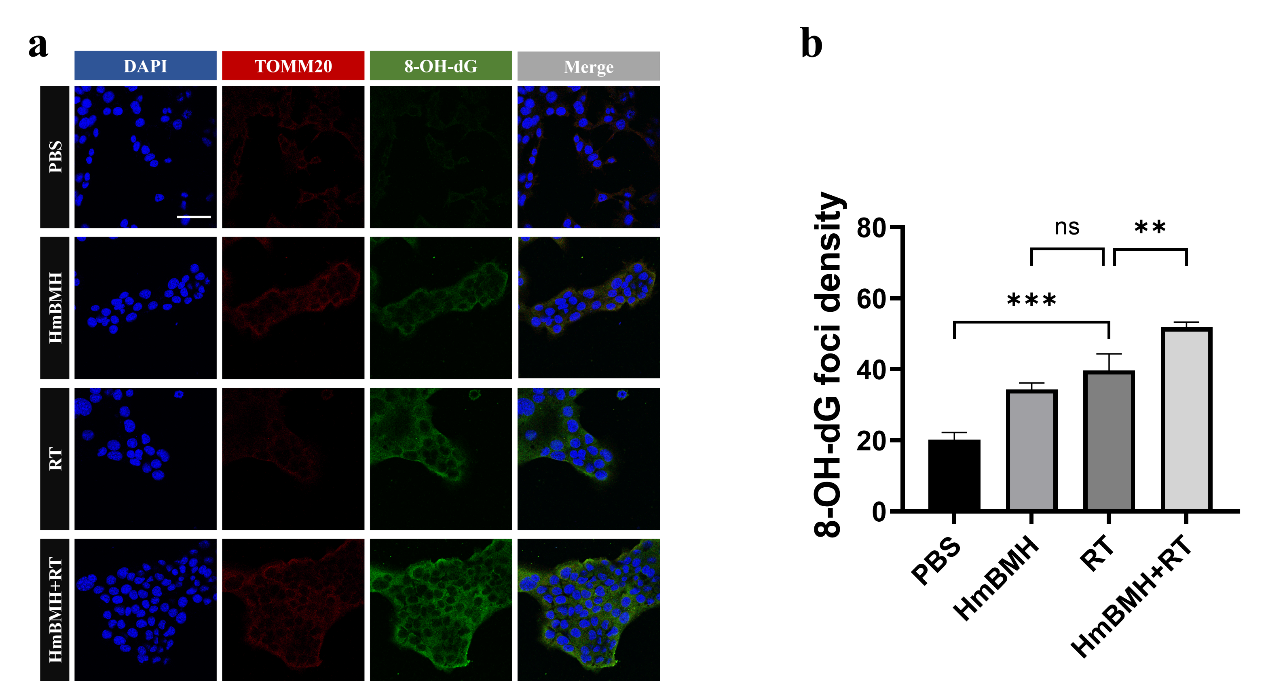


**Figure S13.** (a) Representative immunofluorescence images showing mitoDNA oxidation levels in treated 4T1 cells stained with TOMM20 (red), 8-OHdG (green), and DAPI (blue), scale bar: 50 μm. (b) Quantitative analysis of 8-OH-dG foci density. (n = 3)


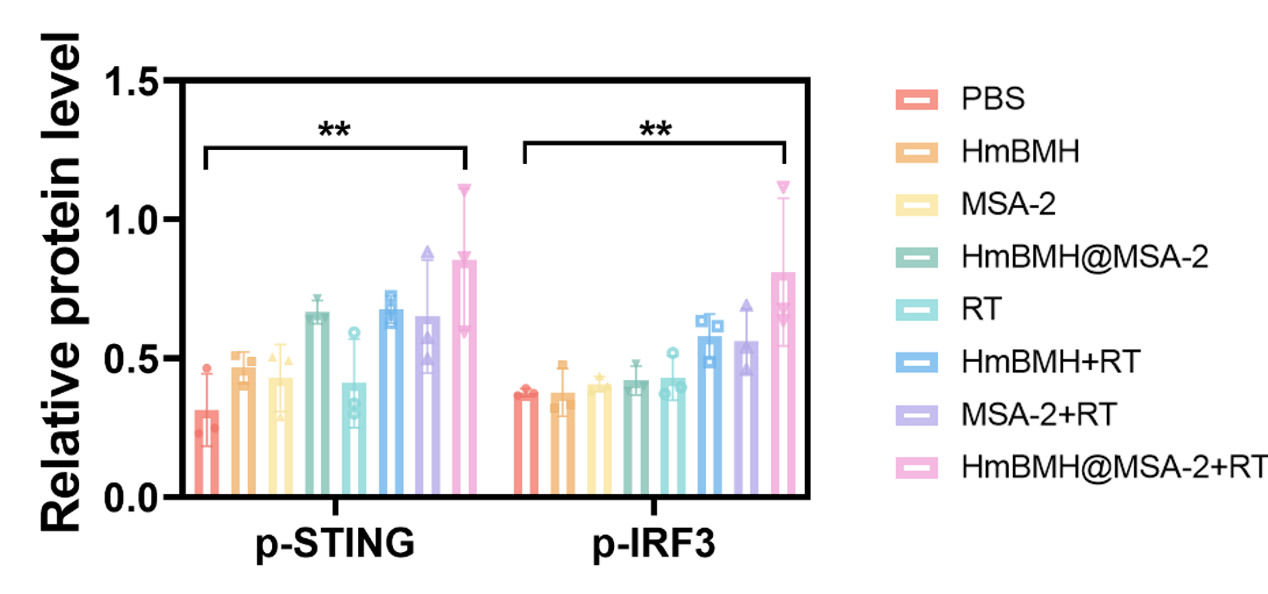


**Figure S14**. The expression levels of p-STING and p-IRF3 in 4T1 cells pretreated with various preparations (n = 3) quantified by Western blot.


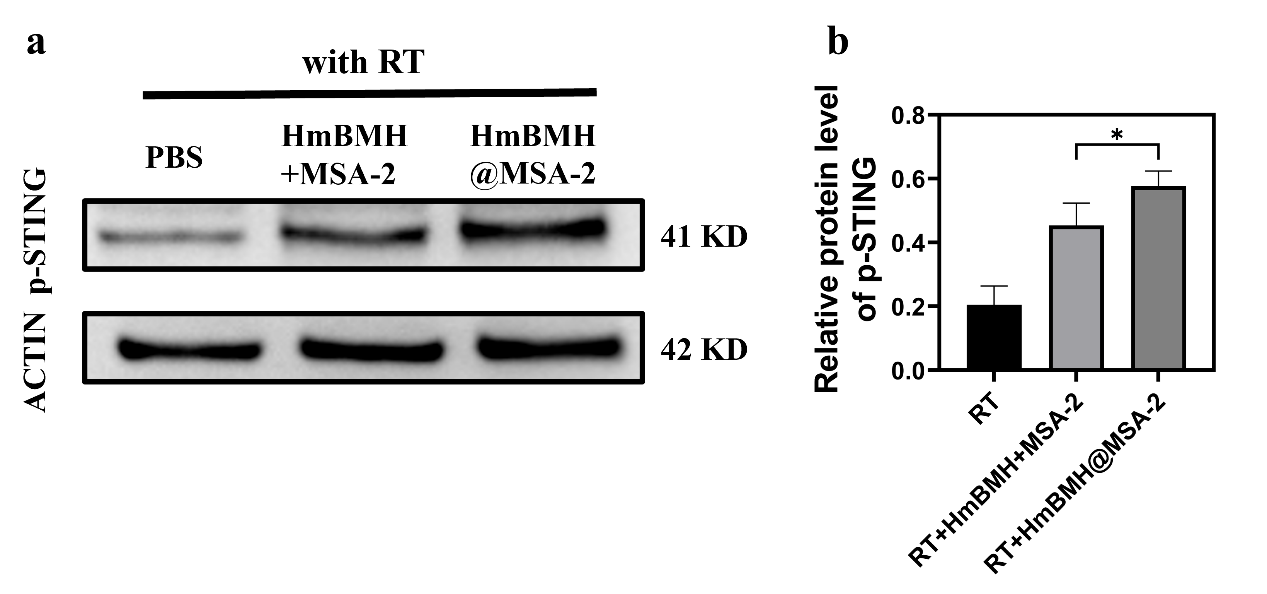


**Figure S15**. (a) Western blot analysis of p-STING expression level in 4T1 cells and (b) their relative quantification. (n = 5)

**
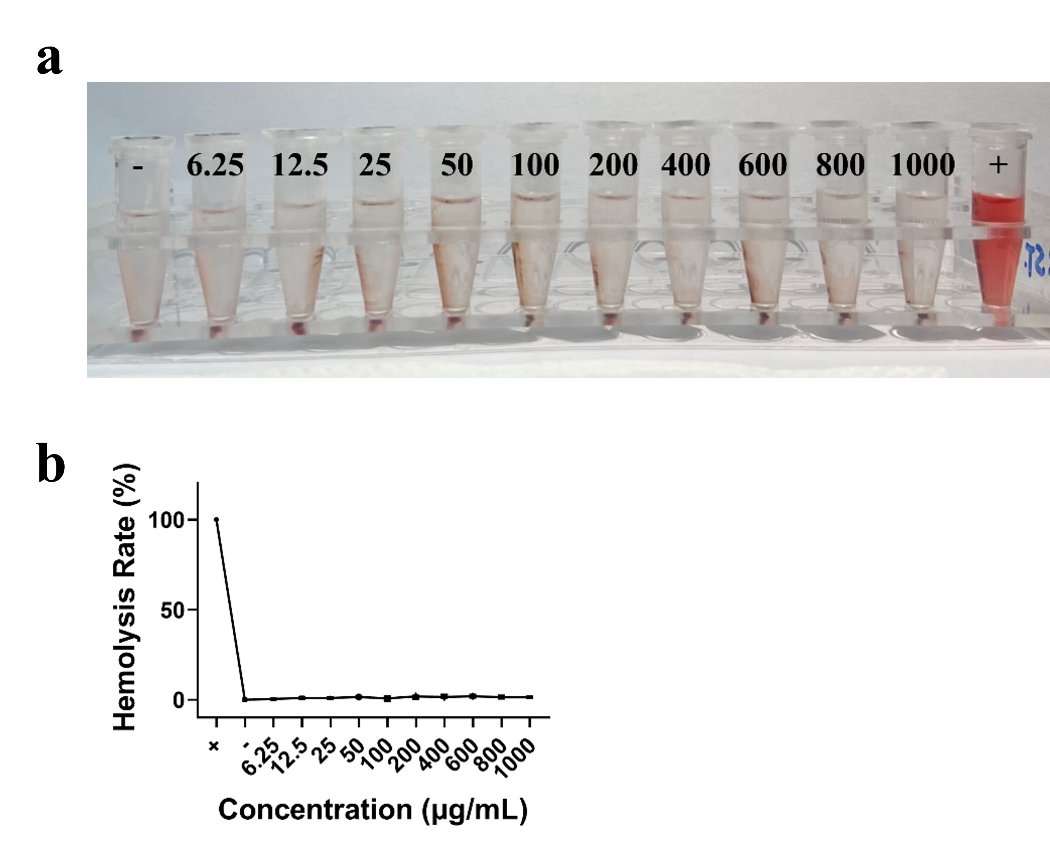
**

**Figure S16**. (a) Hemolysis experiment after incubation with erythrocytes with various concentrations of HmBMH. Deionized water and PBS were used as positive and negative controls, respectively. (b) Quantification of hemolysis for various concentrations of HmBMH. (n = 3)


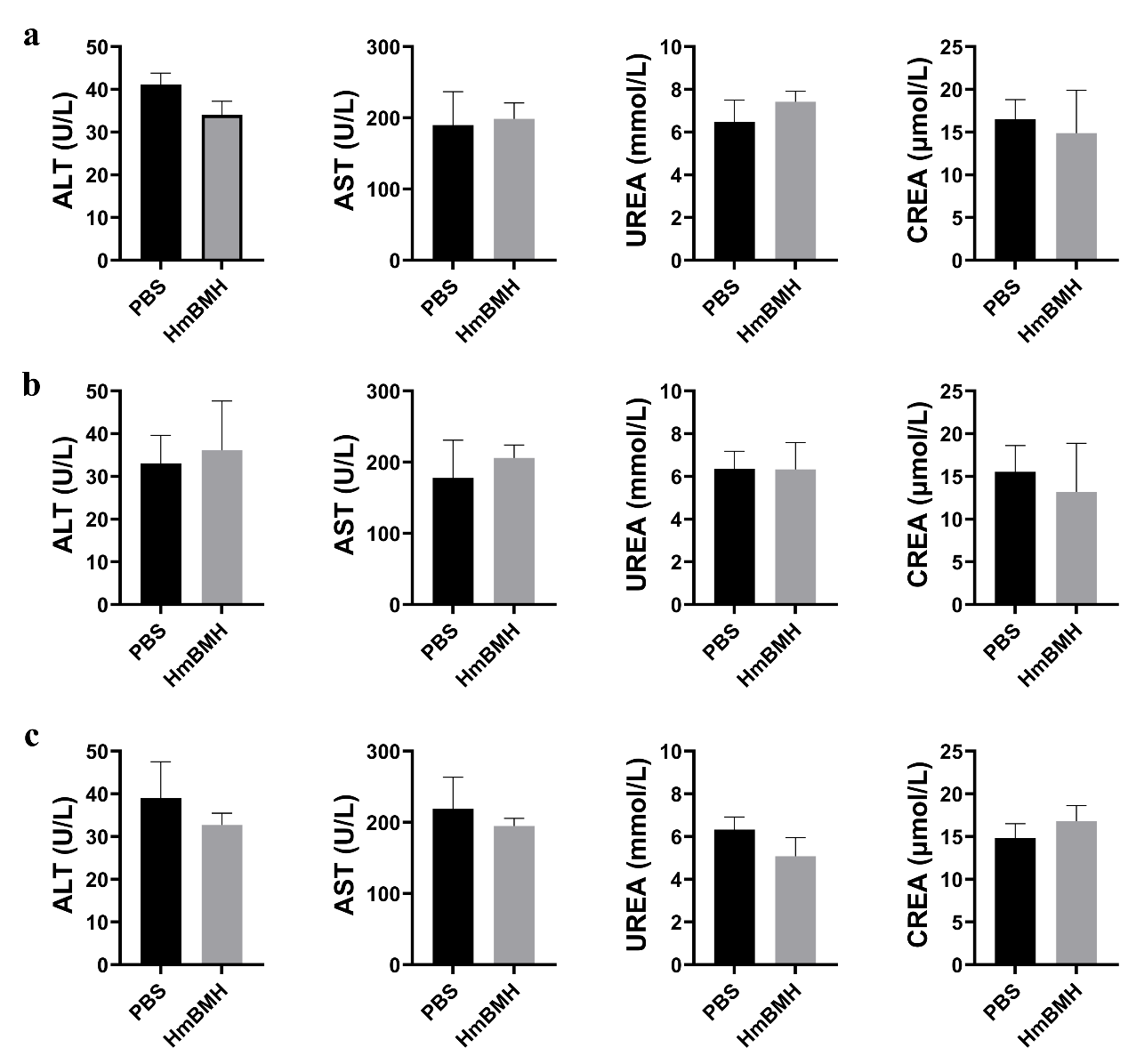


**Figure S17**. Blood biochemical results (ALT, AST, UREA, and CREA) of different treatment groups at (a) 1 d, (b) 14 d, and (c) 28 d. (n = 3)


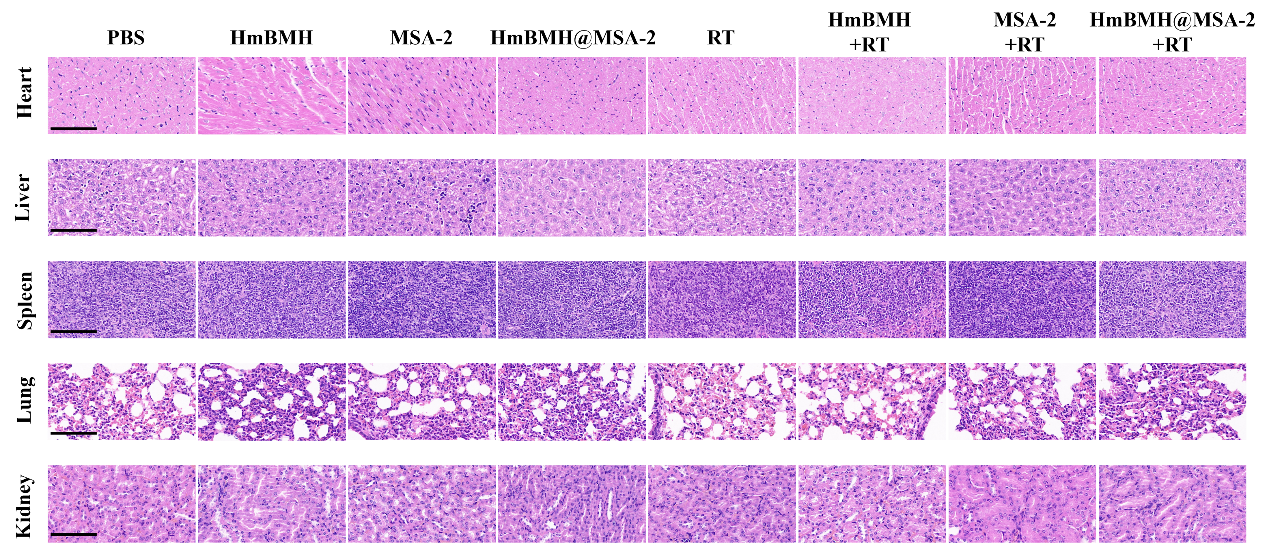


**Figure S18**. Representative images of H&E stained tissue section of the major organs. Scale bar: 100 μm.


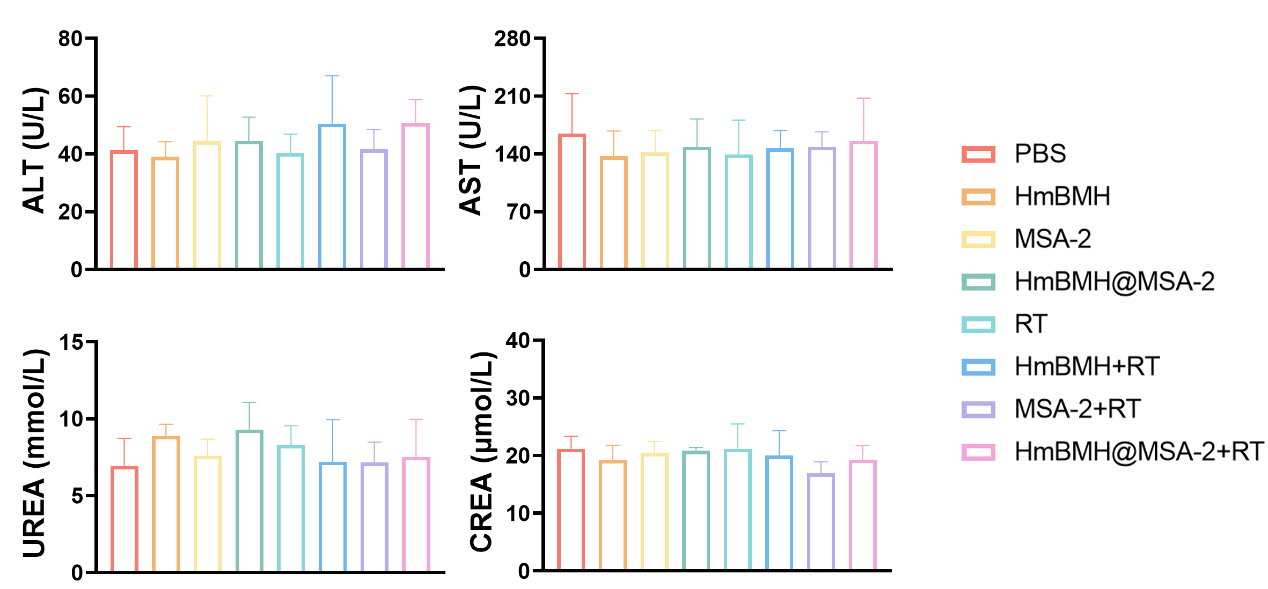


**Figure S19**. Blood biochemical results (ALT, AST, UREA, and CREA) of various treatment groups. (n = 5)


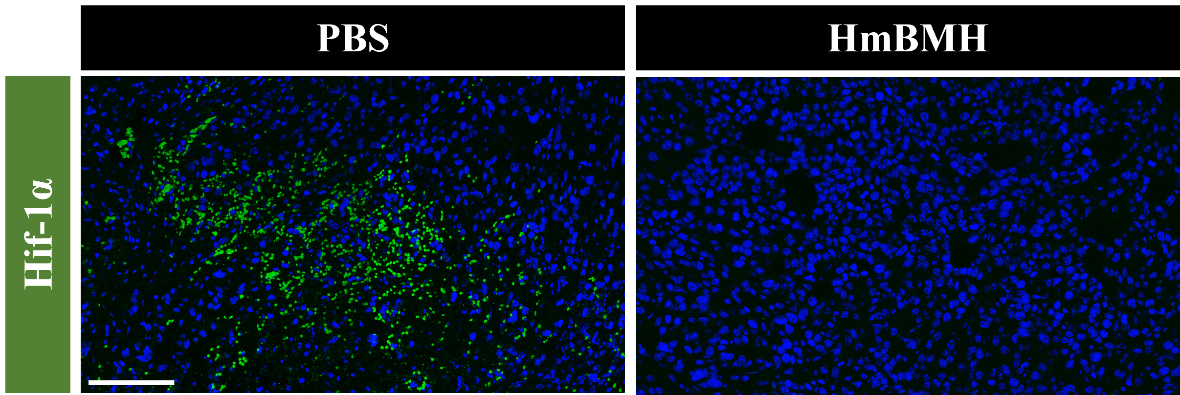


**Figure S20**. Representative immunofluorescence images of HIF-α in the tumor section.


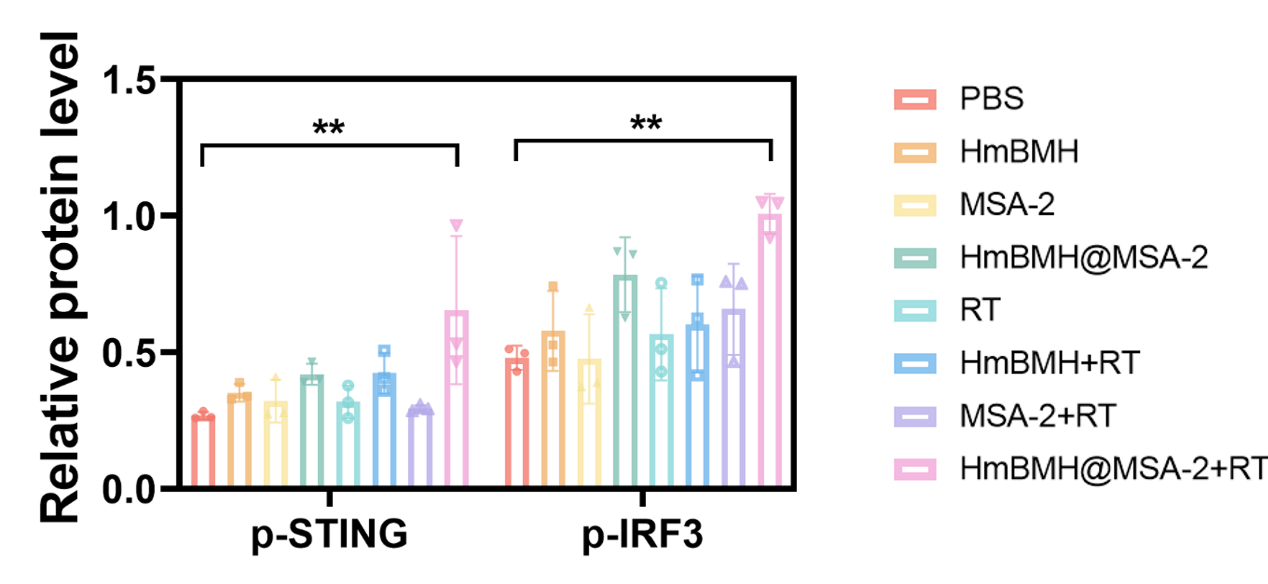


**Figure S21**. The expression levels of p-STING and p-IRF3 in tumor tissue pretreated with various preparations (n = 3) quantified by Western blot.


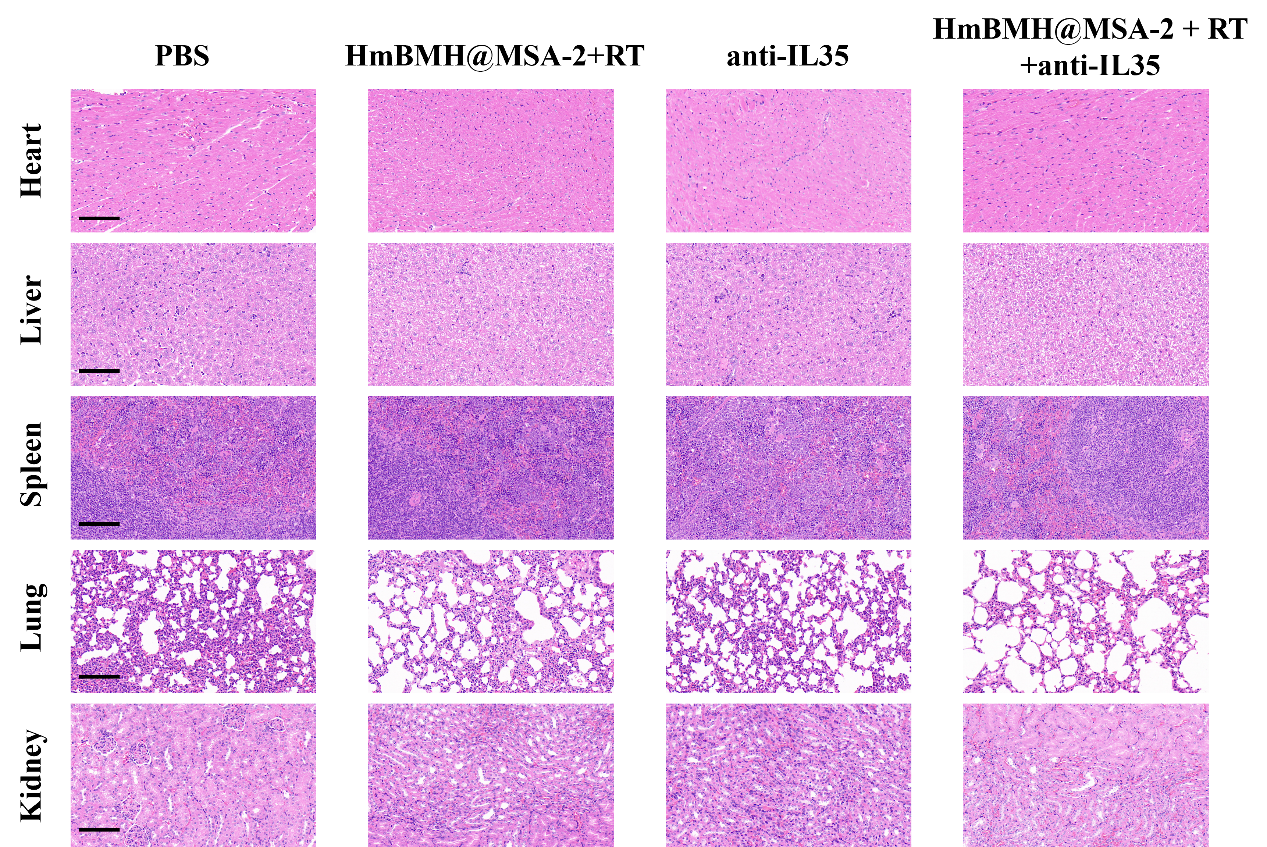


**Figure S22**. Representative images of H&E stained tissue section of the major organs. Scale bar: 20 μm.


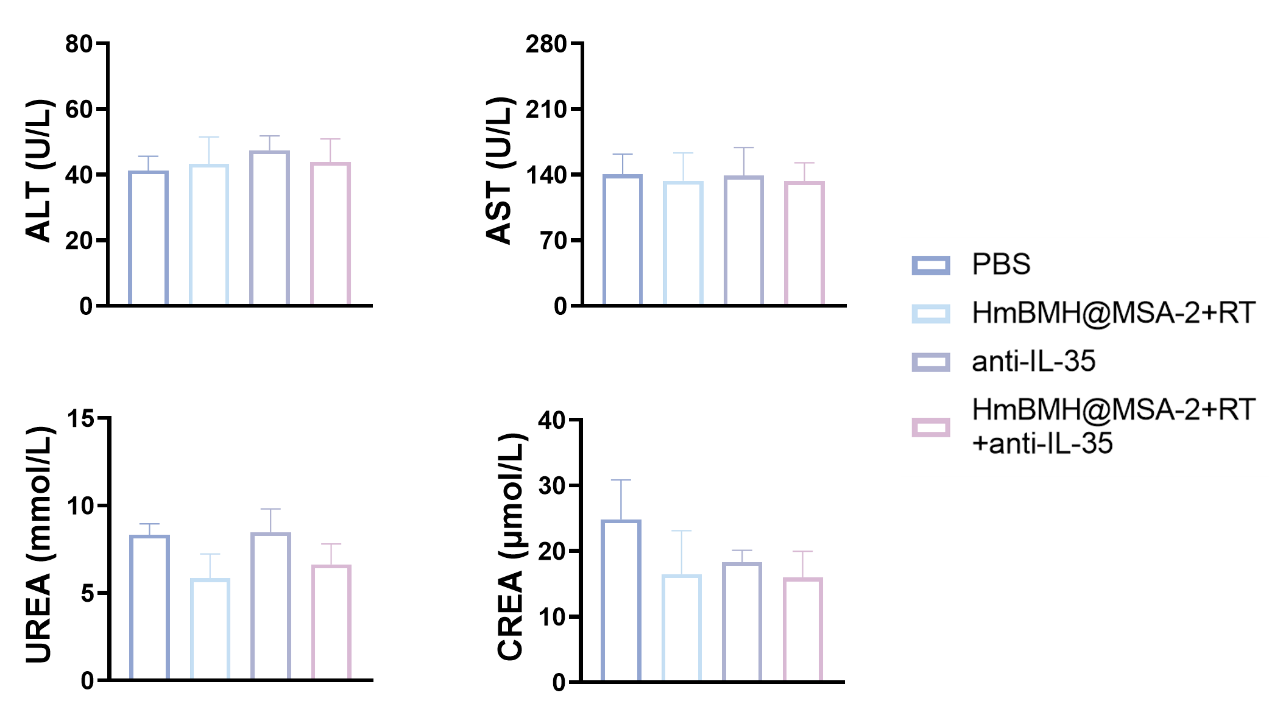


**Figure S23**. Blood biochemical results (ALT, AST, UREA, and CREA) of various treatments groups. (n = 5)


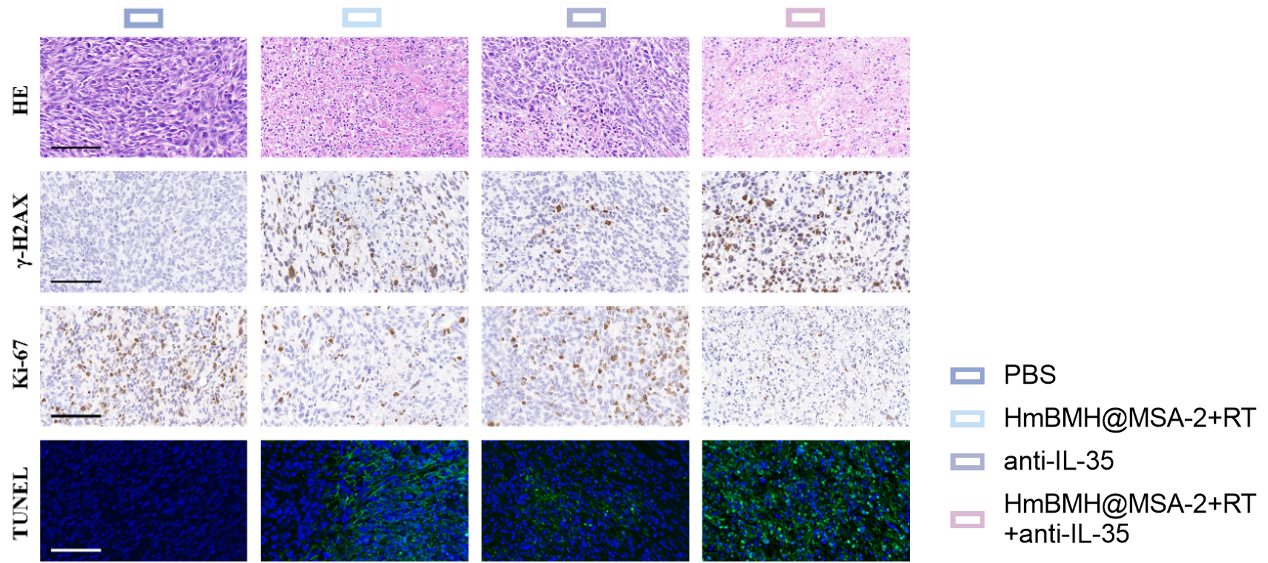


**Figure S24**. H&E, 𝛾-H2AX, Ki-67, and TUNEL staining of the tumors extracted from mice following various treatments, scale bar: 100 μm.


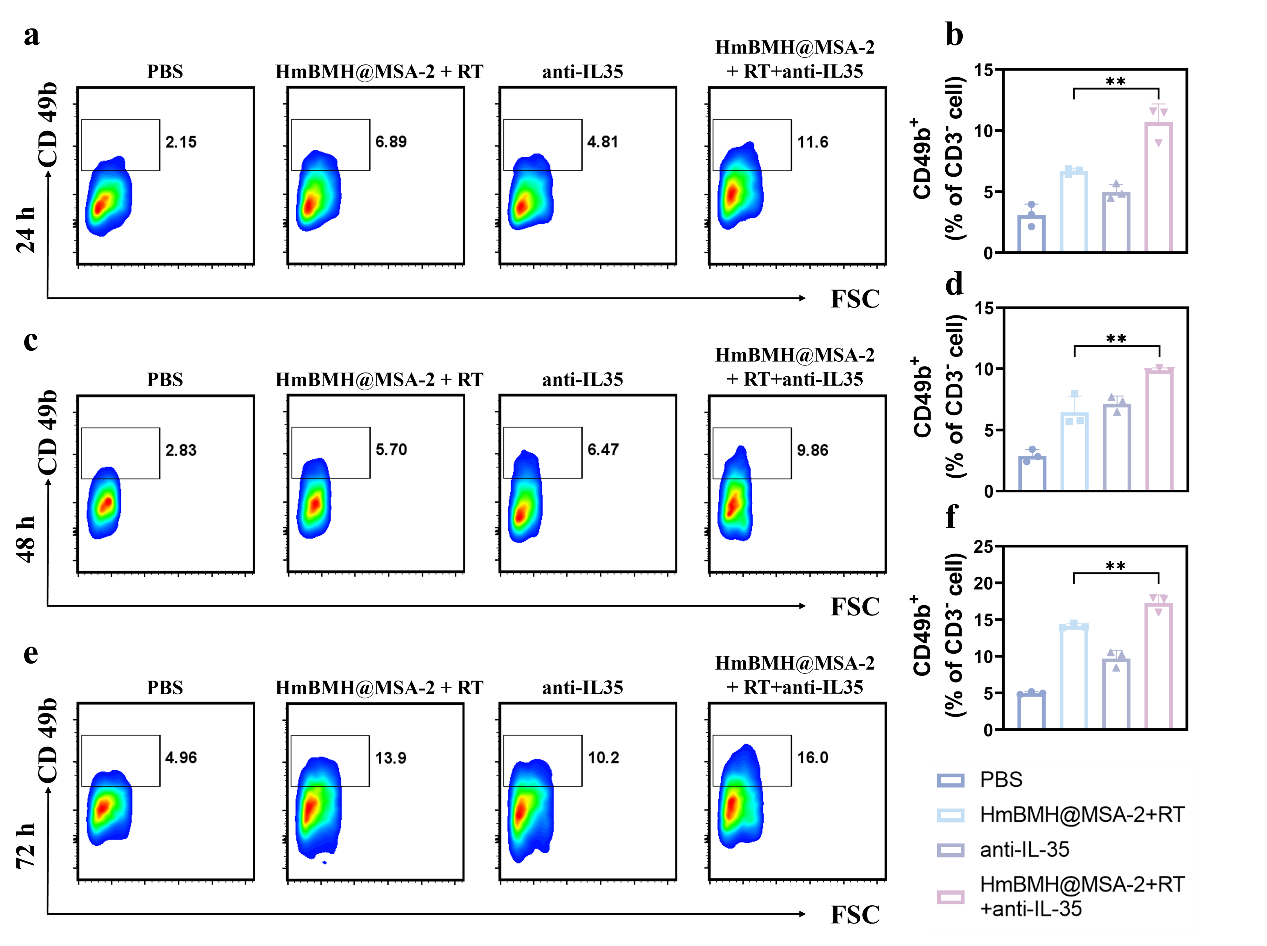


**Figure S25**. Flow cytometry analysis of NK cells in tumor tissues at 24 h (a-b), 48 h (c-d), and 72 h (e-f) from mice with different treatments and the corresponding quantification. (g) Relative NK cell in tumor at different time. (n = 3)


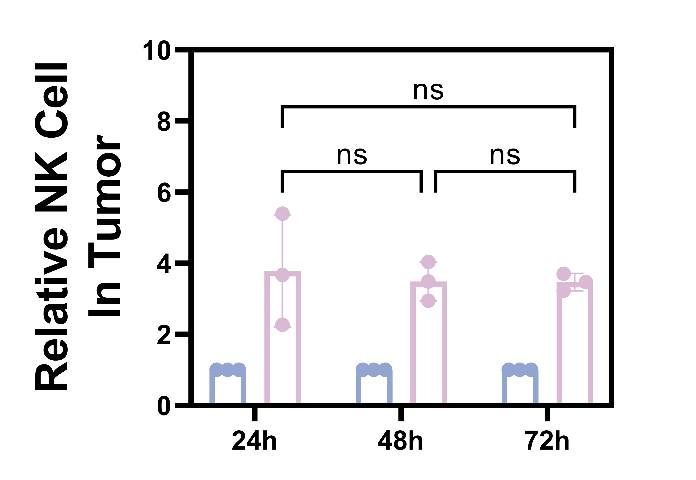


**Figure S26**. Relative NK cell in tumor at different time. (n = 3)


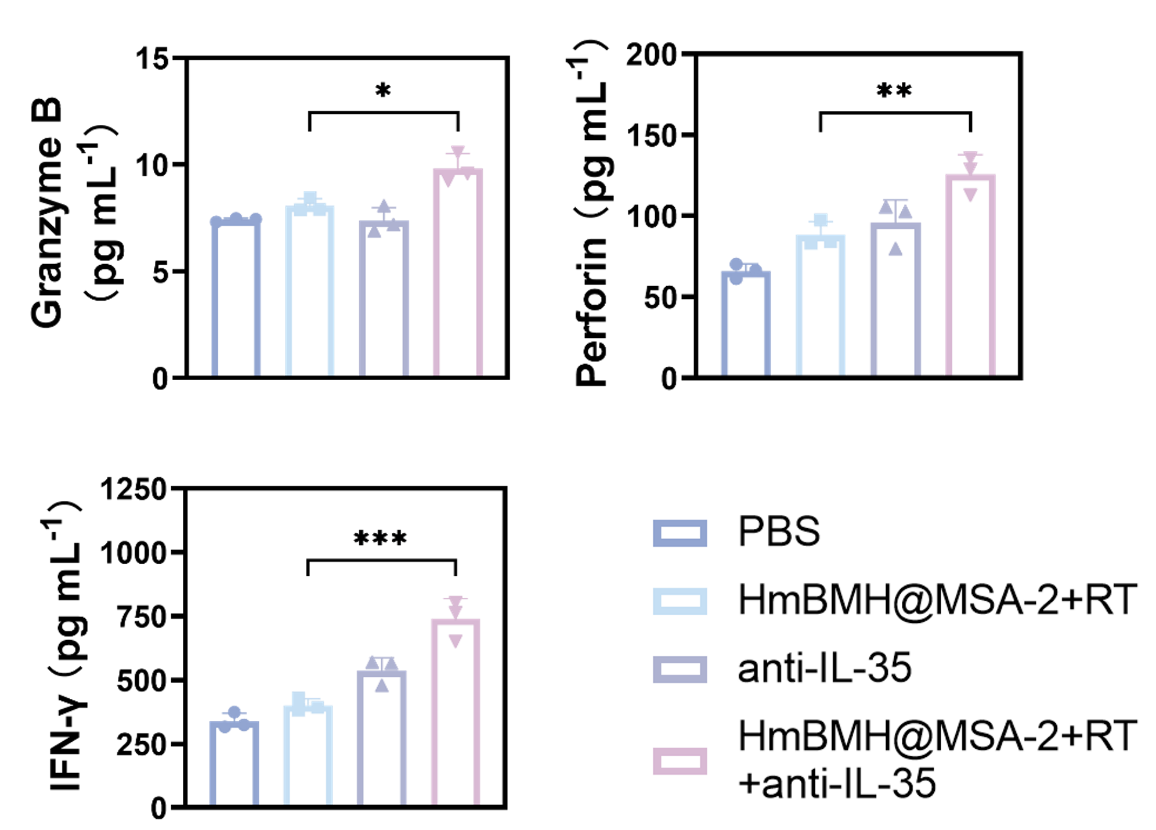


**Figure S27**. ELISA results of proinflammatory cytokine (Granzyme B, Perforin, and IFN-γ) levels in mice serum. (n = 3)


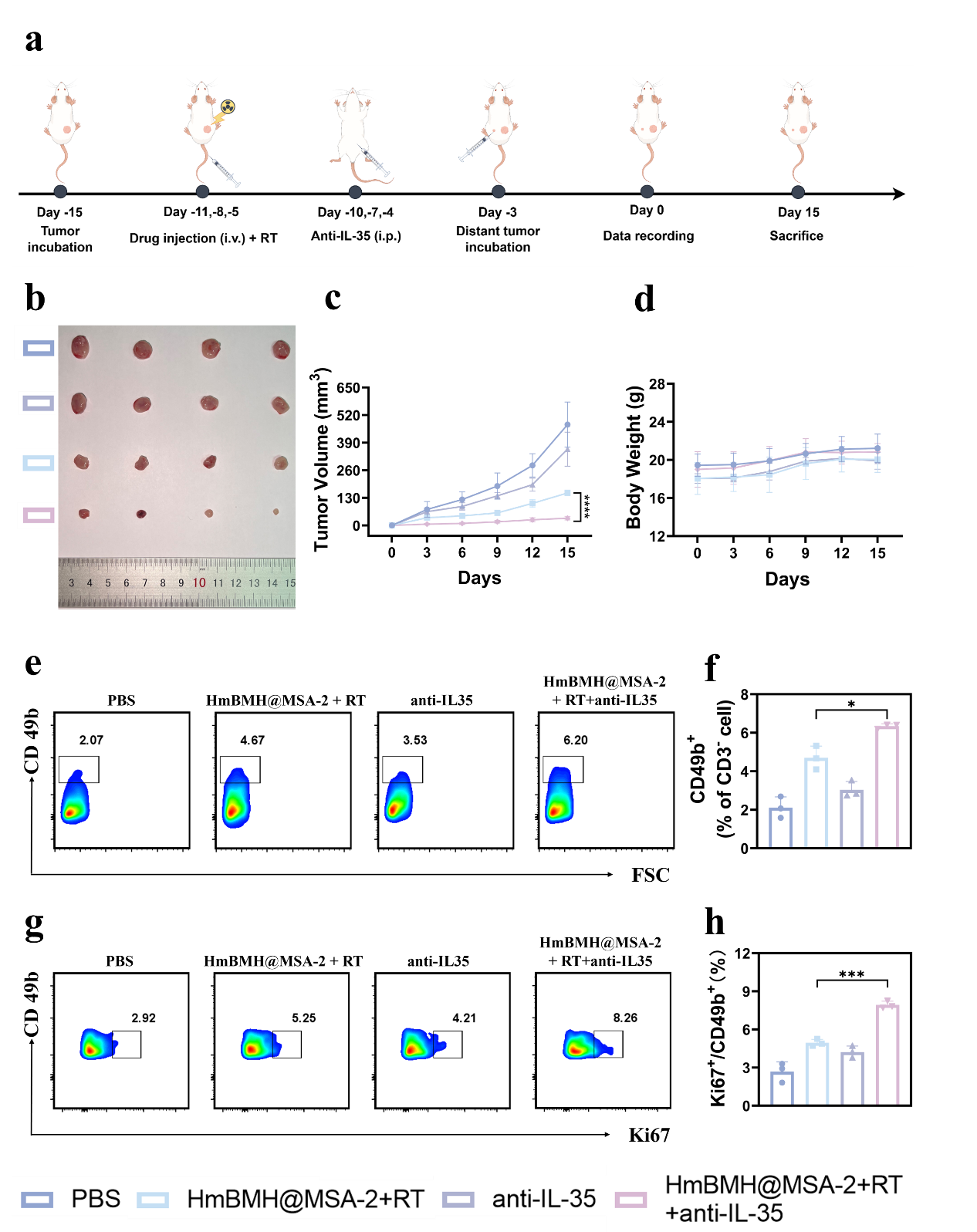


**Figure S28**. (a) Schematic illustration of the distant tumor experiment in combination with anti-IL-35. (b) Photograph of tumors collected from sacrificed mice after different treatments. (c) Relative tumor volume of mice from different groups. (d) Body weight curves of mice from different groups. (e-f) Flow cytometry analysis of NK cells in distant tumor tissues from mice with different treatments and the corresponding quantification. (g-h) Flow cytometry analysis of Ki67-expressing NK cells in distant tumor tissues from mice with different treatments and the corresponding quantification. Data were given as mean ± S.D. (n ≥ 3). Statistical significance was calculated via one-way ANOVA with Tukey’s test: *P < 0.05, ***P <0.001, ****P < 0.0001.
